# ARGONAUTE10 promotes the degradation of miR165/6 through the SDN1 and SDN2 exonucleases in *Arabidopsis*

**DOI:** 10.1371/journal.pbio.2001272

**Published:** 2017-02-23

**Authors:** Yu Yu, Lijuan Ji, Brandon H. Le, Jixian Zhai, Jiayi Chen, Elizabeth Luscher, Lei Gao, Chunyan Liu, Xiaofeng Cao, Beixin Mo, Jinbiao Ma, Blake C. Meyers, Xuemei Chen

**Affiliations:** 1 Department of Botany and Plant Sciences, Institute of Integrative Genome Biology, University of California, Riverside, California, United States of America; 2 Department of Plant and Soil Sciences, and Delaware Biotechnology Institute, University of Delaware, Newark, Delaware, United States of America; 3 State Key Laboratory of Genetic Engineering, Collaborative Innovation Center of Genetics and Development, Department of Biochemistry, Institute of Plant Biology, School of Life Sciences, Fudan University, Shanghai, China; 4 State Key Laboratory of Plant Genomics and National Center for Plant Gene Research, Institute of Genetics and Developmental Biology, Chinese Academy of Sciences, West Lincui Road, Chaoyang District, Beijing, China; 5 Guangdong Provincial Key Laboratory for Plant Epigenetics, College of Life Sciences and Oceanography, Shenzhen University, Shenzhen, China; 6 Howard Hughes Medical Institute, University of California, Riverside, California, United States of America; University of Massachusetts Medical School, UNITED STATES

## Abstract

The degradation of small RNAs in plants and animals is associated with small RNA 3′ truncation and 3′ uridylation and thus relies on exonucleases and nucleotidyl transferases. ARGONAUTE (AGO) proteins associate with small RNAs in vivo and are essential for not only the activities but also the stability of small RNAs. AGO1 is the microRNA (miRNA) effector in *Arabidopsis*, and its closest homolog, AGO10, maintains stem cell homeostasis in meristems by sequestration of miR165/6, a conserved miRNA acting through AGO1. Here, we show that SMALL RNA DEGRADING NUCLEASES (SDNs) initiate miRNA degradation by acting on AGO1-bound miRNAs to cause their 3′ truncation, and the truncated species are uridylated and degraded. We report that AGO10 reduces miR165/6 accumulation by enhancing its degradation by SDN1 and SDN2 in vivo. In vitro, AGO10-bound miR165/6 is more susceptible to SDN1-mediated 3′ truncation than AGO1-bound miR165/6. Thus, AGO10 promotes the degradation of miR165/6, which is contrary to the stabilizing effect of AGO1. Our work identifies a class of exonucleases responsible for miRNA 3′ truncation in vivo and uncovers a mechanism of specificity determination in miRNA turnover. This work, together with previous studies on AGO10, suggests that spatially regulated miRNA degradation underlies stem cell maintenance in plants.

## Author summary

MicroRNAs (miRNAs) are 21–24 nucleotide regulatory RNAs that impact nearly all biological processes in plants and animals. The abundance of miRNAs is determined by their biogenesis and degradation. miRNA degradation is associated with trimming and tailing of their 3′ ends. The relationship between 3′ trimming and 3′ tailing as well as the enzyme(s) responsible for 3′ trimming was unknown in *Arabidopsis*. Mechanisms that protect miRNAs from degradation include 3′ terminal methylation and association with ARGONAUTE (AGO) proteins. In this study, we show that two members of the SMALL RNA DEGRADING NUCLEASE (SDN) family, SDN1 and SDN2, are partly responsible for the miRNA 3′ trimming activities in *Arabidopsis*. We further elucidate the relationship between 3′ trimming and 3′ tailing—miRNAs are first trimmed by SDNs, and the 3′ trimmed-and-unmethylated miRNAs are tailed by the nucleotidyl transferase HESO1. Furthermore, we demonstrate that SDN1 is able to act on AGO1-bound and methylated miRNAs. We also reveal a special function of AGO10, which has the highest affinity for miR165/6 in vivo. We show that AGO10-bound miR165/6 is more susceptible to SDN-mediated degradation than AGO1-bound miR165/6. This study provides a general molecular framework for miRNA degradation and an example of specificity determination in miRNA degradation.

## Introduction

*Arabidopsis ARGONAUTE10* (*AGO10*), also known as *ZWILLE* (*ZLL*) or *PINHEAD* (*PNH*), maintains stem cell homeostasis in the shoot apical meristem (SAM) and floral meristems through repression of miR165/6 activity [1–5]. The conserved miR165/6 family acts through *AGO1* to downregulate the type III homeodomain-leucine zipper genes that are critical for stem cell maintenance, leaf polarity, and vasculature development [6–10]. *AGO10* is expressed in the adaxial side of organ primordia and in the provasculature underneath the SAM to maintain stem cells in the SAM in a non-cell autonomous manner [3,4,11], whereas miR165/6 is restricted to the abaxial side of organ primordia and excluded from the SAM [2,7]. As AGO10 binds miR165/6 with higher affinity than AGO1, it was hypothesized that AGO10, which accumulates in a highly restricted manner in the plant [3,4,11], sequesters miR165/6 to prevent it from repressing its target genes through the ubiquitously present AGO1 protein [5,12].

*AGO10* has also been implicated in repressing the accumulation of miR165/6. In multiple *ago10* loss-of-function mutants, the levels of miR165/6 are moderately increased (to 1.5–2-fold of wild-type levels), as determined by northern blotting with whole seedlings or inflorescences [1,2,5]. Given that *AGO10*-expressing cells constitute only a tiny portion of the tissues used in these studies, the small increase is likely an underestimate for the ability of *AGO10* to repress miR165/6 accumulation. In fact, in situ hybridization revealed that miR165/6, which is normally excluded from the SAM, accumulates in the SAM in *ago10* mutants [2], suggesting that AGO10 not only sequesters miR165/6 but also represses its accumulation. The impact of AGO10 on miR165/6 contrasts the positive effects of AGO1 on the accumulation of microRNAs (miRNAs), including miR165/6 [1,13]. The mechanism by which AGO10 reduces the levels of miR165/166 is currently unknown.

The steady-state levels of miRNAs are determined by the balance between biogenesis and degradation. miRNA biogenesis is a multistep process. After the transcription of *MIR* genes into pri-miRNAs, DICER-LIKE1 (DCL1) processes pri-miRNAs into pre-miRNAs and pre-miRNAs into the miRNA/miRNA* duplexes. The duplexes are methylated by HEN1, and the miRNA strand is loaded into AGO1, the major miRNA effector, to form the RNA-induced silencing complex (RISC; reviewed in [14]).

The mechanisms of miRNA degradation are not well understood. Degradation intermediates are hard to detect in the wild-type background, but they are readily detectable in *hen1* mutants, in which miRNAs and small interfering RNAs (siRNAs) in plants and Piwi-interacting RNAs (piRNAs) in animals lose 2′-*O*-methylation on the 3′ terminal ribose and are more susceptible to degradation [15–25]. Studies of the consequences of loss of methylation in both plant and animal *hen1* mutants revealed two molecular processes associated with small RNA degradation, namely 3′ truncation and 3′ uridylation [15,18,19,21,22]. The enzyme that causes miRNA 3′ truncation is presumably an exonuclease, but its nature is as yet unknown in *Arabidopsis*. Two nucleotidyl transferases, HESO1 and URT1, play a major and minor role, respectively, in miRNA uridylation [26–29]. Both enzymes are able to uridylate AGO1-bound, unmethylated miRNAs in vitro, and they act in a partially redundant and synergistic manner to uridylate unmethylated miRNAs in vivo [26–29]. The sequence of events in miRNA degradation (truncation followed by tailing or tailing followed by truncation) is unknown.

In *Arabidopsis*, the SMALL RNA DEGRADING NUCLEASE (SDN) family of 3′ to 5′ exonucleases consisting of five family members degrades short RNAs in vitro and limits the accumulation of miRNAs in vivo [30]. Prior in vitro enzymatic assays with SDN1 were performed with free RNA oligonucleotides as substrates, and, in these assays, SDN1 was able to reduce the size of its substrate RNA to a uniform and very small size [30]. This is apparently inconsistent with SDN1 being responsible for the observed miRNA 3′ truncation activity in vivo, as the truncated species lack a small and varying number of nucleotides from the 3′ end. However, in vivo, miRNAs are associated with, and protected by, AGO1; it is possible that the observed varying degree of 3′ truncation is due to the balance between protection by AGO1 and exonucleolytic degradation by SDN1. It is unknown whether SDN1 is able to act on AGO1-bound miRNAs and, if so, whether the interplay between AGO1 and SDN1 leads to the truncation of a varying number of nucleotides. The answer to this question is critical in understanding how miRNAs are degraded in vivo, as SDN1 can act on methylated miRNAs [30] whereas HESO1 and URT1 cannot [26–29], which makes SDN proteins the prime candidates in initiating the degradation of methylated miRNAs in vivo.

In this study, we show that SDN1 and SDN2 are responsible for the 3′ truncation of a subset of miRNAs in the *hen1* background and miR165/6 species in the wild-type background, thereby revealing an enzyme associated with the 3′ truncation process in vivo. We show that 3′ truncated miRNAs are further tailed by HESO1 to lead to their degradation, thus clarifying the relationship between the two miRNA degradation processes. Furthermore, we show that, in vitro, SDN1 acts on AGO1-bound, methylated miRNAs to produce 3′ truncated miRNAs of varying sizes, similar to those observed in vivo. These findings provide a molecular framework of miRNA degradation that acts on many miRNAs. Furthermore, we show that AGO10 promotes the degradation of its associated miR165/6 in vivo, and this effect requires SDNs. AGO10-bound miR165/6 is more susceptible to SDN1-mediated 3′ truncation than AGO1-bound miR165/6 in vitro. This study reveals an unexpected activity of an AGO protein, uncovers a mechanism of specificity determination in miRNA turnover, and implicates the importance of regulated miRNA degradation in stem cell maintenance.

## Results

### SDN1 and SDN2 are responsible for the 3′ truncation of some miRNAs in vivo

In wild type, 3′ truncated miRNA species are rare, presumably because they are rapidly degraded. Thus, to determine whether SDNs are responsible for miRNA 3′ truncation, we resorted to a *hen1* mutant, in which the lack of 3′ terminal 2′-*O*-methylation of miRNAs is associated with rampant miRNA 3′ truncation and 3′ uridylation. Truncated and/or tailed species of miRNAs are readily detectable by northern blotting [22,25] and quantifiable by small RNA high throughput sequencing (sRNA-seq) [26–29]. To ascertain whether SDNs are responsible for the production of 3′ truncated miRNA species in vivo, we generated the *hen1-8 sdn1-1 sdn2-1* triple mutant (hereafter referred to as *hen1 sdn1 sdn2*) and compared its miRNA profiles with those of the *hen1-8* single mutant by sRNA-seq. To determine the sequence of events (3′ truncation versus 3′ tailing), we also examined published sRNA-seq data from *hen1-8* and *hen1-8 heso1-1* [29]. Reads corresponding to each miRNA were classified into the full-length (FL), 3′ truncated-only (TR-only), 3′ tailed-only (TA-only), and 3′ truncated-and-tailed (TR+TA) categories and quantified [29]. For both pairs of genotypes, two biological replicates were performed or analyzed. To be consistent, we present the 23 most abundant miRNAs (reads per million (RPM) > 10) across all eight libraries.

**Fig 1:**
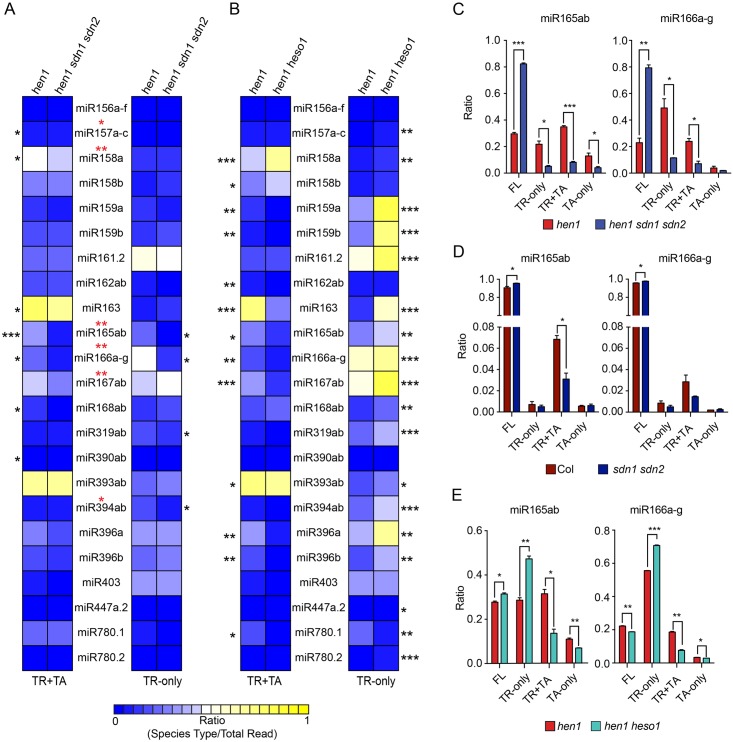
SDN1 and 2 are responsible for miRNA 3′ truncation in vivo. High-throughput sequencing was conducted to profile miRNAs in wild type (Col), *sdn1 sdn2*, *hen1*, and *hen1 sdn1 sdn2*. Published sRNA-seq from *hen1* and *hen1 heso1* [29] was also analyzed. Two biological replicates were performed for each genotype, and the error bars represent standard deviations. * *p* < 0.05, ** *p* < 0.01, *** *p* < 0.001. (A-B) Heatmaps showing the levels of TR+TA and TR-only species of 23 miRNAs in *hen1 sdn1 sdn2* (A) or *hen1 heso1* (B) as compared to *hen1*. For each miRNA, the ratio of TR+TA (or TR-only) species to all reads corresponding to this miRNA is shown. The red asterisks indicate statistically significant reduction in the sum of TR+TA and TR-only species for the indicated miRNAs (no miRNAs showed a statistically significant increase). (C-D) The composition of miR165 and miR166 reads corresponding to FL, TR-only, TR+TA, and TA-only species in *hen1*, *hen1 sdn1 sdn2*, Col, and *sdn1 sdn2*, as indicated. (E) The composition of miR165 and miR166 reads in *hen1* and *hen1 heso1*. The reduction in the levels of TR+TA species in *hen1 heso1* is accompanied by the increase in the levels of TR-only species. Underlying data can be found in the Gene Expression Omnibus (GEO) database as series GSE35479 and GSE58138. The raw data for panels (A-E) can be found in S1 Data.

We compared the levels of TR-only and TR+TA species in *hen1 sdn1 sdn2* and *hen1* as these species represented the miRNA 3′ truncation activity. Nine out of the 23 miRNAs showed a significant reduction in the levels of either TR+TA or TR-only species in *hen1 sdn1 sdn2* (Fig 1A, S2 Data, and S1 Fig). In addition, one miRNA (miR167ab) showed a significant reduction in TR+TA+TR-only species, although the reduction in either TR+TA or TR-only species was not statistically significant (Fig 1A). Thus, ten of the 23 miRNAs showed reduced 3′ truncation. Many miRNAs also showed reduced 3′ truncation in both biological replicates but did not pass the *p*-value cutoff (<0.05), while few miRNAs showed increased 3′ truncation (S2 Data). This indicates that *SDN1* and *SDN2* are responsible for the production of 3′ truncated species in vivo from at least some miRNAs. Functional redundancy with the remaining family members could be responsible for the lack of an observable effect on the 3′ truncation of other miRNAs. Alternatively, non-SDN exonucleases also cause miRNA 3′ truncation.

Having shown that SDN1 and SDN2 cause the 3′ truncation of some miRNAs, we next sought to determine which occurred first, truncation by SDN1/2 or tailing by HESO1. Either is theoretically possible—SDN1 truncates miRNAs and HESO1 uridylates truncated miRNAs, or HESO1 uridylates miRNAs and SDN1 acts on uridylated miRNAs to cause their 3′ truncation. In the *hen1 heso1* double mutant, the levels of TR+TA species for many of the 23 miRNAs were reduced, and those of TR-only species were increased (Fig 1B and S3 Data). Therefore, HESO1 tailed the TR-only species to generate the TR+TA species. In particular, for the ten miRNAs with a significant reduction in 3′ truncation in *hen1 sdn1 sdn2* (Fig 1A), nine showed a significant increase in TR-only species in *hen1 heso1* (Fig 1B). These data indicate that 3′ truncated species generated by SDNs are further uridylated by HESO1 for degradation.

miR165/6 species were drastically affected in *hen1 sdn1 sdn2* relative to *hen1*. The proportion of FL species of miR165 and miR166 was much higher in *hen1 sdn1 sdn2* than in *hen1*, while those of TR-only or TR+TA species were much reduced (Fig 1C and S1 Fig). The proportion of TA-only species was either unaffected (miR166) or affected to a smaller extent (miR165; Fig 1C and S1 Fig). This demonstrated a role of SDN1 and SDN2 in the production of 3′ truncated species of miR165/6 in the *hen1* background. In the wild-type background, the proportions of TR-only, TR+TA, and TA-only miR165/6 species were much lower than those in the *hen1* background (Fig 1C and 1D). Nevertheless, a reduction in the proportions of TR+TA species was observed in *sdn1 sdn2* relative to wild type (Fig 1D). Therefore, SDN1 and SDN2 are responsible for the 3′ truncation of miR165/6 in vivo in both *hen1* and wild-type backgrounds. In *hen1 heso1*, the reduction in TR+TA miR165 or miR166 species is accompanied by an increase in TR-only species (Fig 1E), indicating that HESO1 tails 3′ truncated miR165/6 species generated by SDN1/2.

### SDN1 acts on AGO1-bound, methylated miRNAs in vitro

Given the results above, an appealing model (S2 Fig, right panel) of miRNA degradation is that methylated, FL miRNAs are first truncated by SDN1/2, which results in 3′ truncated miRNAs that lack 3′ terminal methylation. These 3′ truncated species are then tailed by HESO1 and URT1 to cause their complete degradation. One important question related to this model is whether SDN1/2 can act on AGO1-bound miRNAs (S2 Fig, left panel), as mature miRNAs are associated with AGO1 in vivo. HESO1 and URT1 are able to uridylate AGO1-bound, unmethylated miRNAs in vitro [26–29], but previous biochemical assays with SDN1 were only conducted with free RNA oligonucleotides as substrates [30]. Intriguingly, although SDN1 degrades RNA substrates to a uniform and very small size in vitro [30], the 3′ truncated species that depended on SDN1/2 for accumulation in vivo had a small and varying number of nucleotides truncated from the 3′ ends (S1 Fig). One possibility is that SDN1 cannot completely degrade AGO1-bound miRNAs but, instead, only cause their 3′ truncation.

**Fig 2:**
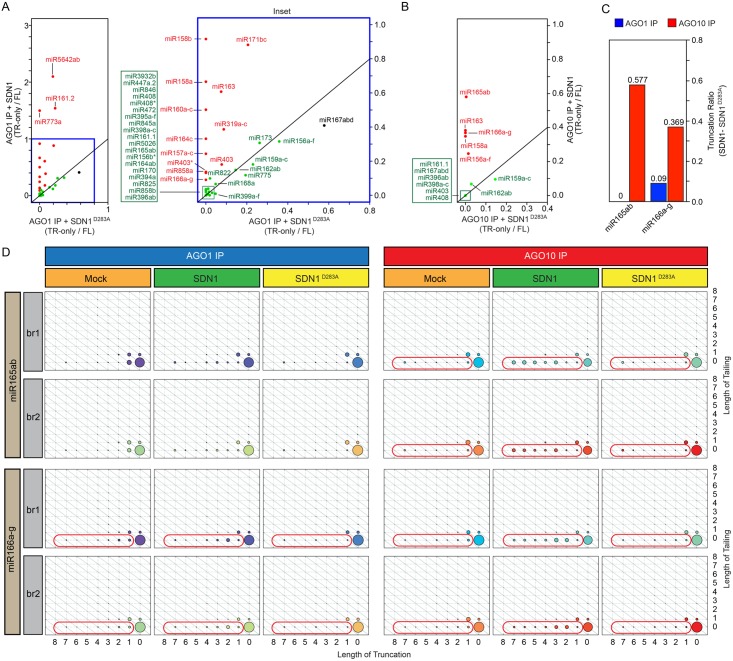
SDN1 is able to trim AGO1- and AGO10-bound miRNAs in vitro. AGO1 IP or AGO10 IP was used as the substrate in enzymatic reactions with mock (no enzyme), SDN1, or a catalytic mutant (SDN1^D283A^) in two biological replicates. sRNA-seq was conducted to examine the 3′ trimming of miRNAs. (A-B) Plots showing the ratio of 3′ TR-only species to FL species for various miRNAs after the enzymatic reactions. TR-only species present in the mock control were subtracted from the SDN1 or SDN1^D283A^ reactions. The diagonal lines represent equal levels of 3′ truncated species in the SDN1 and SDN1^D283A^ reactions, indicating that the truncated species were not generated by SDN1. The miRNAs marked in red showed SDN1-mediated 3′ truncation. As the levels of 3′ truncation were low, the two biological replicates were combined to derive the values. Only abundant miRNAs (RPM > 10) were examined. (C) Bar plot showing higher levels of 3′ truncation by SDN1 of miR165/6 in AGO10 IP than in AGO1 IP. The *y*-axis represents SDN1-mediated 3′ truncation; any truncated species present in the SDN1^D283A^ reactions were subtracted from those in the SDN1 reactions. (D) Diagrams showing the status of 3′ truncation and tailing of miR165/6. The *x*-axis represents the number of nucleotides truncated from the 3′ end. The *y*-axis represents the number of nucleotides added to the 3′ end. The relative proportions of the species are indicated by the sizes of the circles. Two biological replicates (br1 and br2) are shown separately. The 3′ truncated species accumulating at higher levels in the SDN1 reactions relative to the mock and SDN1^D283A^ reactions are marked. Underlying data can be found in the GEO database as series GSE87355. The raw data for panels (A-C) can be found in S1 Data.

To test this, we conducted SDN1 assays with AGO1 immunoprecipates (IPs; S3A Fig) as the substrate under enzyme excess conditions. miR165/6 was detected by northern blotting before and after the reactions. While SDN1 was able to nearly completely degrade a free RNA oligonucleotide, it was largely ineffective in degrading miR165/6 in AGO1 IP (S3B Fig). Thus, AGO1 protects miR165/6 from being degraded by SDN1 in vitro. However, we noticed that upon extended incubation (>2 hr), weak signals representing shorter miR165/6 species were detectable in the AGO1 IP (S3B Fig), suggesting that SDN1 caused miR165/6 3′ truncation at a low level. As northern blotting was not a sensitive method to detect such 3′ truncated species, we performed sRNA-seq to determine whether SDN1 caused 3′ truncation of AGO1-bound miR165/6 and other miRNAs. AGO1 IP was used as the substrate in assays with a mock (no enzyme) control, SDN1, and a catalytic mutant (SDN1^D283A^) control for 1 hr. After the reactions, AGO1 was precipitated again, and the associated small RNAs were subjected to high throughput sequencing. Two biological replicates yielded highly similar results (S4 Fig and S4 Data). For the quantification of miRNA 3′ truncation, the 3′ truncated species present in the mock reactions were subtracted from those in the SDN1 or SDN1^D283A^ reactions. We analyzed the 3′ truncation status of all abundant miRNAs (RPM > 10 in all six samples). Fifteen of 43 abundant miRNAs exhibited higher levels of 3′ truncation in the SDN1 reactions as compared to the SDN1^D283A^ reactions (Fig 2A). Several conclusions can be drawn from this in vitro study. First, SDN1 can act on AGO1-bound miRNAs, and, unlike its activities on free RNAs, it generates heterogeneous, 3′ truncated species from AGO1-bound miRNAs (Fig 2D), consistent with the 3′ truncation observed in *hen1* mutants in vivo. Second, miRNAs in AGO1 IP should be methylated. Thus, SDN1 can act on methylated, AGO1-bound miRNAs, consistent with its ability to degrade methylated RNA oligonucleotides [30]. Third, SDN1 was ineffective against many AGO1-bound miRNAs in vitro (Fig 2A), suggesting that other factors assist SDN1 in miRNA 3′ truncation in vivo or that other exonucleases also cause miRNA 3′ truncation in vivo.

### *AGO10* represses miR165/6 accumulation

**Fig 3:**
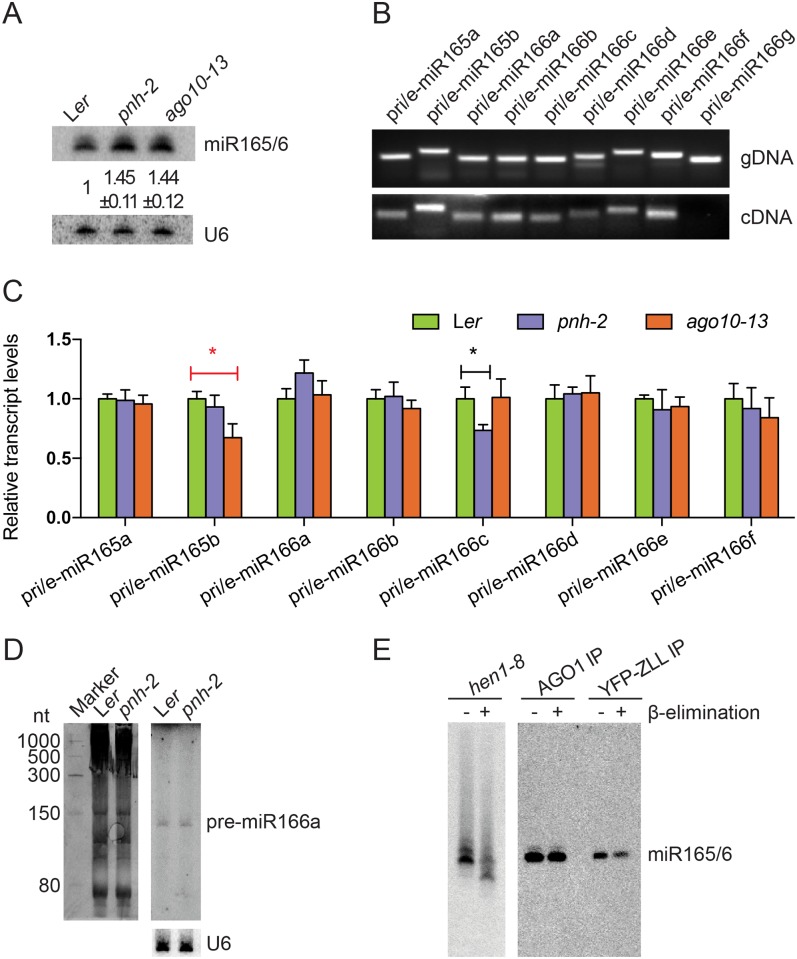
*ago10* mutations do not affect miR165/6 biogenesis. (A) Mature miR165/6 levels were increased in the seedlings of *pnh-2* and *ago10-13* as compared to wild type (L*er*) in six biological replicates. The numbers represent mean ± standard deviations. (B) PCR to test the specificity of primers from each *MIR165/6* locus, using genomic DNA as the template (upper panel); RT-PCR to determine whether the nine *MIR165/6* genes were expressed (lower panel). Reverse transcription was conducted with random primers followed by PCR with the same pairs of gene-specific primers as used for the PCR with genomic DNA above. The primers were designed such that the sum of pri- and pre-miR165/6 species (denoted by “pri/e-miR165/6”) from a *MIR165/6* gene was detected. (C) Real-time RT-PCR to measure the relative transcript levels of pri/e-miR165/6s in wild type (L*er*) versus *pnh-2* and *ago10-13* seedlings. * *p* < 0.05 (D) The levels of pre-miR166a in wild type (L*er*) versus *pnh-2* as determined by northern blotting. The image on the left was the stained gel. U6 was a loading control. (E) AGO1- or AGO10-bound miR165/6 is methylated. AGO1 and AGO10 were separately immunoprecipitated from *zll-1 ZLLp*::*YFP-ZLL* plants, and the associated RNAs were subjected to β-elimination followed by northern blotting to examine the methylation status of miR165/6. The lack of a shift in molecular weight after β-elimination indicated that miR165/6 was fully methylated. A control (*hen1-8*) was included to show that the β-elimination treatment was effective, as the miRNA lacking methylation in this mutant showed an expected shift. Note that the *hen1-8* RNA was treated at the same time as the AGO IPs, resolved in the same gel, and transferred to the same membrane. The membrane was split into two for northern blotting to prevent the signals from the *hen1-8* sample from interfering with the signals in the AGO IPs. The raw data for panel C can be found in S1 Data.

Loss-of-function *ago10* mutants, such as *pnh-2* and *ago10-13*, show increased levels of miR165/6 [1,2]. In northern blots with young seedlings of wild type, *pnh-2* and *ago10-13*, we consistently observed an increase in miR165/6 levels in six biological replicates (Fig 3A). We examined whether the increase in miR165/6 accumulation in these mutants could be attributed to enhanced miR165/6 biogenesis. miR165/6 is encoded by two *MIR165* and seven *MIR166* loci. We designed primers that allowed the detection of the sum of pri- and pre-miRNA species from each of the nine loci. Reverse transcription PCR (RT-PCR) was performed to determine whether pri/pre-miR165/6 species from all nine loci were present in young seedlings in wild type. While PCR, using genomic DNA as the template, produced a specific band at each locus, RT-PCR produced a band at all nine loci except for *MIR166g* (Fig 3B). The finding that eight *MIR165/6* genes were expressed in young, wild-type seedlings was in agreement with findings from analyses of promoter activities of *MIR165/6* genes [31]. We performed real-time RT-PCR in wild-type, *pnh-2*, and *ago10-13* seedlings for these eight loci. The levels of the eight pri/pre-miR165/6 species were not increased in the two *ago10* mutants (Fig 3C). Consistent with this analysis, northern blotting showed that the levels of pre-miR166a were similar in wild-type and *pnh-2* seedlings (Fig 3D). Therefore, loss of function in *AGO10* resulted in an increase in the levels of mature miR165/6 but did not affect the transcription of *MIR165/6* genes or the processing of pri-miR165/6, suggesting that *AGO10* represses miR165/6 accumulation at a step after precursor processing.

AGO10 has been shown to recognize features of the miR165/miR165* or miR166/miR166* duplex during the loading of miR165/6 into AGO10 [5]. As miRNA/miRNA* duplexes are the substrates of HEN1, we asked whether AGO10’s association with the duplex of miR165/6 and miR165/6* could compete with HEN1-mediated methylation. We immunoprecipitated AGO1 and AGO10 from a *zll-1 ZLLp*::*YFP-ZLL* line in which the *YFP-ZLL* (*AGO10*) transgene driven by the *ZLL* (*AGO10*) promoter fully rescues the morphological defects of *zll-1* [11].

β-elimination assays that interrogated the methylation status of miR165/6 showed that both AGO1- and AGO10-bound miR165/6 species were fully methylated in vivo (Fig 3E), suggesting that *AGO10* does not affect the methylation status of this miRNA. Therefore, we conclude that *AGO10* must repress the accumulation of miR165/6 at a step after its biogenesis.

### *AGO10* overexpression causes a reduction in miR165/6 levels

**Fig 4:**
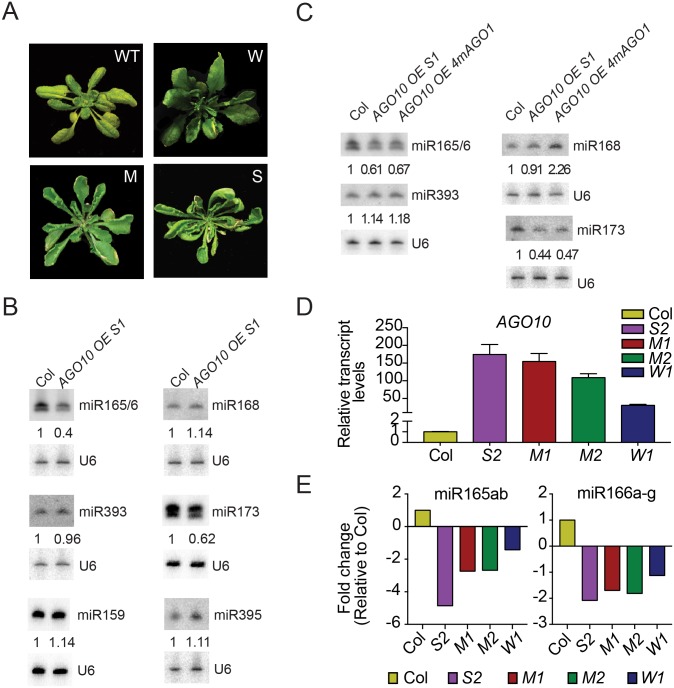
*AGO10* overexpression causes reduced miR165/6 accumulation. (A) Phenotypes of wild type (WT) and *AGO10* overexpression (*AGO10 OE*) lines in Weak (W), Moderate (M), and Strong (S) categories. The classification of *AGO10 OE* lines was based on the degree of deviation from the wild-type leaf phenotype. (B) The levels of six miRNAs in wild-type and *AGO10 OE S1* seedlings, as determined by northern blotting. U6 served as the internal control, and the numbers indicate the abundance of the miRNAs in *AGO10 OE S1* relative to wild type (Col). (C) The levels of four miRNAs in wild-type, *AGO10 OE S1*, and *AGO10 OE 4mAGO1* seedlings, as determined by northern blotting. U6 served as the internal control, and the numbers indicate the abundance of the miRNAs relative to wild type (Col). (D) Real-time RT-PCR to determine *AGO10* transcript levels in wild type (Col) and four *AGO10 OE* lines. (E) Levels of miR165/6 in various *AGO10 OE* lines as compared to wild type (Col). The abundance of the miRNAs was determined by sRNA-seq. Underlying data can be found in the GEO database as series GSE87355. The raw data for panels (D-E) can be found in S1 Data.

*AGO10* is expressed in a highly restricted manner in meristems and developing organ primordia, and the expression domains of *AGO10* and miR165/6 are largely exclusive [2–4,7,11]. We reasoned that, if AGO10’s association with miR165/6 leads to the degradation of the miRNA, *AGO10* overexpression and ectopic expression should lead to further sequestration of miR165/6 from AGO1 and, consequently, a reduction in miR165/6 levels. To test this hypothesis, we introduced *YFP-AGO10*, driven by the strong and constitutive Cauliflower Mosaic Virus 35S promoter into wild-type plants. Among independent T1 transgenic lines, most exhibited phenotypic alterations similar to what was previously observed to be associated with *AGO10* overexpression [32]. The phenotypes were classified into the Weak, Moderate, and Strong categories (Table 1). Plants in the different categories were largely similar in size to wild-type plants, but they differed from wild type and from each other in the degrees of leaf hyponasty (upward curling) and serration, as shown in Fig 4A. We focused on an *AGO10* overexpression (*AGO10 OE*) line in the Strong category (referred to as *AGO10 OE S1*) for subsequent analyses. *AGO10* mRNA levels were much higher in this line than in wild type (S5A Fig). A large reduction in miR165/6 levels was observed in *AGO10 OE S1* (Fig 4B). Levels of pri/pre-miR165/6 from the eight genes with detectable expression in seedlings were unaffected (S5B Fig). The levels of pre-miR166a were also unaffected by *AGO10* overexpression in two independent transgenic lines (one in the wild-type background [*AGO10 OE S1*] and the other in *pnh-2*; S5C Fig). These data indicated that *AGO10* overexpression did not affect the biogenesis of miR165/6.

**Table 1:** *sdn1 sdn2* partially suppresses the developmental phenotype of *AGO10 OE*.

	*AGO10 OE*	*sdn1 sdn2 AGO10 OE*
WT-like	9 (25.7%)	21 (36.8%)
Weak	4 (11.4%)	25 (43.9%)
Moderate	5 (14.3%)	6 (10.5%)
Strong	17 (48.6%)	5 (8.8%)
**Total**	35	57

Developmental phenotypes of the primary *AGO10 OE* transformants were classified into four categories: wild type—like, Weak, Moderate, and Strong, based on the degree of deviation from the wild-type leaf phenotype. Thirty-five primary *AGO10 OE* transformants in wild-type background and 57 *AGO10 OE* transformants in the *sdn1 sdn2* background were analyzed.

Abbreviations: WT, wild type.

Among the six miRNAs examined by northern blotting in *AGO10 OE S1*, miR173 was found to also accumulate at a lower level (Fig 4B). This raised the possibility that the reduced abundance of miR165/6 and miR173 in *AGO10 OE S1* was due to reduced *AGO1* expression, as previous studies demonstrated the association between miR168 and AGO10 and implicated *AGO10* in the repression of *AGO1* expression at the posttranscriptional level through miR168 [1,33]. Real-time RT-PCR showed that the levels of *AGO1* mRNA were reduced by about 20% in *AGO10 OE S1* (S5A Fig). AGO1 protein levels were reduced by about 50% (S5D Fig). To evaluate whether *AGO10* overexpression indirectly repressed the accumulation of miR165/6 and miR173 through inhibition of *AGO1* expression, we introduced *4mAGO1*, which renders *AGO1* resistant to miR168 [13], into *AGO10 OE S1*. Transgenic lines were screened by RT-PCR to obtain one in which *AGO10* mRNA levels were comparable to those of *AGO10 OE S1*, but *AGO1* expression was elevated (S5E Fig). AGO1 protein levels were also elevated in *AGO10 OE 4mAGO1* (S5F Fig). Elevated *AGO1* expression in *AGO10 OE 4mAGO1* failed to rescue the levels of miR165/6 or miR173 (Fig 4C). Note that miR168 levels were comparable in wild type and *AGO10 OE S1* and elevated in *AGO10 OE 4mAGO1* (Fig 4B and 4C), consistent with the previous observation that miR168 accumulation is tightly buffered by AGO1 [34]. In conclusion, although *AGO10* overexpression led to reduced *AGO1* expression, the reduced accumulation of miR165/6 and miR173 was not attributable to lower *AGO1* expression.

We evaluated whether there were any dosage effects of *AGO10* overexpression on miR165/6 levels. We chose another four independent lines of *AGO10 OE* based on the severity of the morphological phenotypes (Fig 4A). Lines S2, M1 and M2, and W1 were from the Strong, Moderate, and Weak categories, respectively. The levels of *AGO10* mRNA in these lines were concordant with the severity of morphological defects (Fig 4D). We conducted sRNA-seq for wild type and the four *AGO10 OE* lines (S5 Data). The abundance of miR165 and miR166 was reduced in all four lines and was largely anticorrelated with the levels of *AGO10* expression (Fig 4E).

### *AGO10* overexpression results in increased 3′ truncation of miR165/6

**Fig 5:**
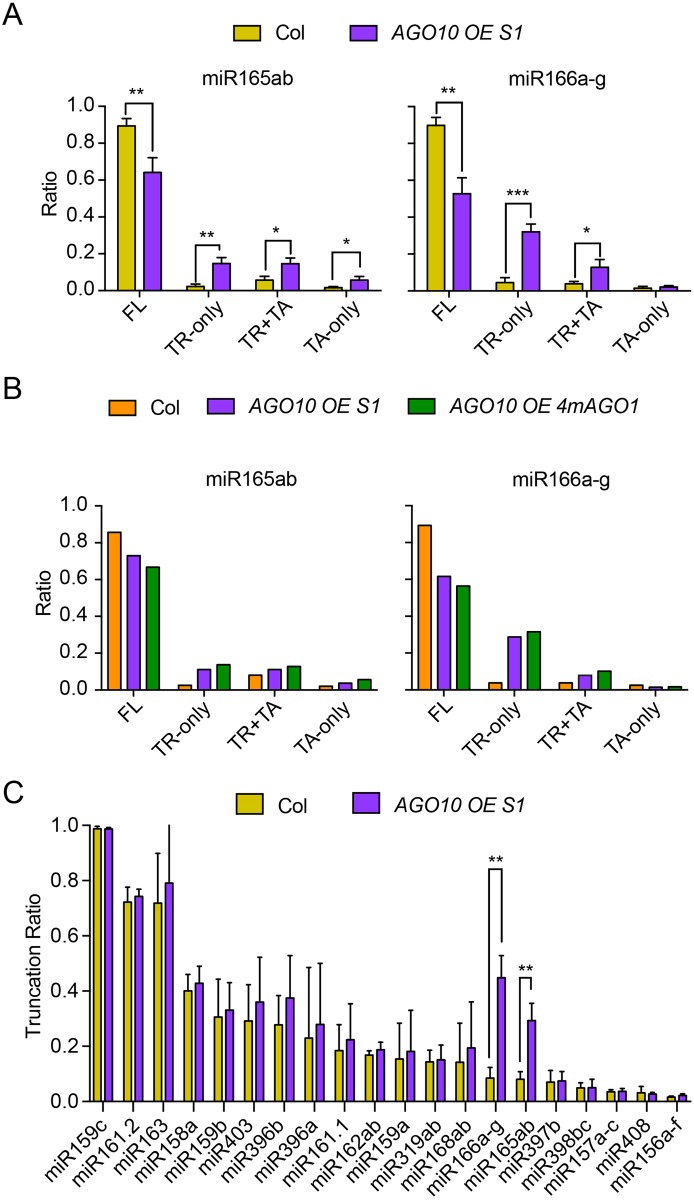
*AGO10* overexpression results in reduced levels of FL and increased levels of 3′ truncated miR165/6. High-throughput sequencing was conducted to profile miRNAs in wild type (Col) and *AGO10 OE S1*. Three biological replicates were performed, and the error bars represent standard deviations, * *p* < 0.05, ** *p* < 0.01, *** *p* < 0.001. One replicate was performed for *AGO10 OE 4mAGO1*. (A) Proportions of various types of miR165/6 species in wild type (Col) and *AGO10 OE S1*. (B) Proportions of various types of miR165/6 species in wild type (Col), *AGO10 OE S1*, and *AGO10 OE 4mAGO1*. *4mAGO1* failed to suppress the elevated 3′ truncation of miR165/6 in *AGO10 OE*. (C) The status of 3′ truncation for the 20 most abundant miRNAs in the sRNA-seq datasets. Truncation ratio is the proportion of truncated species (TR+TA and TR-only) in total species for each miRNA. Underlying data can be found in the GEO database as series GSE58138. The raw data for panels (A-C) can be found in S1 Data.

As *AGO10* loss-of-function and overexpression led to higher and lower levels of miR165/6, respectively, without affecting miR165/6 biogenesis, we hypothesized that *AGO10* promotes miR165/6 degradation. As miRNA degradation is manifested by the presence of 3′ truncated and/or 3′ tailed intermediates, we examined the status of miRNA 3′ truncation and tailing in wild type and *AGO10 OE S1* with sRNA-seq (three biological replicates). The proportion of FL miR165/6 reads was significantly reduced in *AGO10 OE S1* (Fig 5A, S6 Fig and S6 Data). Species of miR165/166 showing 3′ truncation, including both TR-only and TR+TA forms, were increased in *AGO10 OE* relative to wild type, suggesting that *AGO10* overexpression enhanced the 3′-to-5′ truncation of miR165/6 (Fig 5A, S6 Fig and S6 Data). Of the TA-only species, only miR165 was modestly increased in abundance (Fig 5A and S6 Data). This suggested that 3′ truncation, but not 3′ tailing, was the primary event in miR165/6 degradation induced by *AGO10 OE*. To examine whether the enhanced degradation of miR165/6 in *AGO10 OE* was due to reduced *AGO1* expression, we also sequenced small RNAs from *AGO10 OE 4mAGO1* plants. The 3′ truncation of miR165/6 induced by *AGO10* overexpression was not rescued by *4mAGO1* (Fig 5B, S6 Fig, and S7 Data). Therefore, the degradation of miR165/6 induced by *AGO10* overexpression was not attributable to reduced *AGO1* expression.

We examined whether *AGO10* overexpression affected the status of 3′ truncation of other miRNAs. For the top 20 most abundant miRNAs in the sRNA-seq datasets (Col and *AGO10 OE S1*), miR165/6 were the only species with significant changes in 3′ truncation (Fig 5C). No miRNAs showed reduced 3′ truncation.

### *AGO10* overexpression causes further sequestration of miR165/6 from AGO1

**Fig 6:**
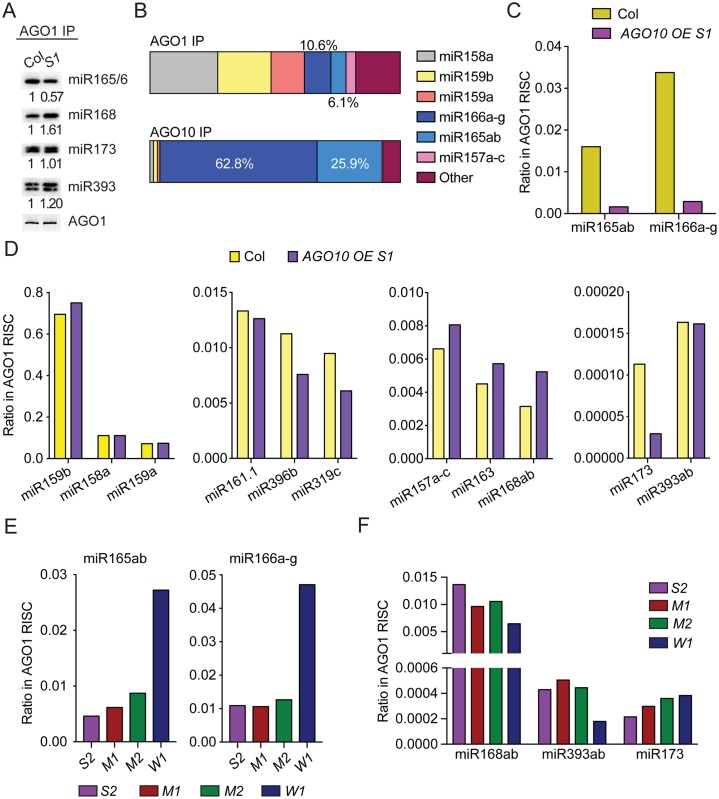
*AGO10* overexpression results in sequestration of miR165/6 from AGO1. (A) AGO1 was immunoprecipitated from wild type (Col) and *AGO10 OE S1*, and the associated RNAs were subjected to northern blotting to detect four miRNAs. The AGO1 IPs were also subjected to western blotting to determine the levels of the AGO1 protein. (B) Composition of miRNAs in AGO1 and AGO10 IP from *AGO10 OE S1*. sRNA-seq was performed with three biological replicates of AGO1 and AGO10 IP from *AGO10 OE S1*. Reads corresponding to a particular miRNA were quantified against those of all annotated miRNAs and shown as percentage of total reads. (C-D) The levels of various miRNAs in AGO1 IP from wild type (Col) and *AGO10 OE S1*. The *y*-axis indicates the proportion of an individual miRNA in the total miRNA pool in AGO1 IP. A few miRNAs from each of four magnitudes of abundance levels in AGO1 IP (as indicated by the *y*-axis scales) were chosen to be shown in (D); the choices were random except for miR168, miR173, and miR393, which were chosen for comparison with (A). The data were derived from one biological replicate. (E-F) Levels of various miRNAs in AGO1 IP from various *AGO10 OE* lines with varying levels of *AGO10* expression (in decreasing order from S2 to W1). The *y*-axis indicates the proportion of an individual miRNA in the total miRNA pool in AGO1 IP. Data were from one biological replicate. Underlying data can be found in the GEO database as series GSE58138 and GSE87355. The raw data for panel B and panels (C-F) can be found in S8 and S1 Data, respectively.

To elucidate how *AGO10* overexpression repressed miR165/6 accumulation, we first tested whether *AGO10* overexpression caused sequestration of miR165/6 from AGO1. AGO1 was immunoprecipitated from wild-type and *AGO10 OE S1* seedlings, and four associated miRNAs were examined by northern blotting. Note that AGO1 levels in *AGO10 OE S1* were about 50% of those in wild type (S5D Fig), but for northern blotting to detect AGO1-associated miRNAs, the amounts of AGO1 IP were adjusted such that AGO1 levels were similar in the two genotypes (Fig 6A). Relative to a similar amount of AGO1, miR165/6 was the only miRNA with reduced levels in AGO1 IP from *AGO10 OE S1* (Fig 6A). Thus, *AGO10* overexpression caused further sequestration of miR165/6 from AGO1.

To obtain a global picture of the miRNAs associated with AGO1 and AGO10 in *AGO10 OE S1*, we performed sRNA-seq from AGO1 and AGO10 IPs. Three biological replicates were performed for AGO1 and AGO10 IP from *AGO10 OE S1*. The binding of AGO1 (or AGO10) to a miRNA was quantified by the percentage of reads corresponding to this miRNA in total reads for all annotated miRNAs identified within the small RNA library from the AGO1 (or AGO10) IPs. Results showed that the overall profiles of miRNAs associated with AGO1 or AGO10 in *AGO10 OE* *S1* resembled those in wild type [5]. AGO1 associated with most miRNAs while AGO10 preferentially associated with miR165/6 (Fig 6B and S8 Data).

To determine whether *AGO10* overexpression caused a further sequestration of miR165/6 from AGO1, AGO1 IP was performed with wild type and *AGO10 OE S1* with one biological replicate. Consistent with the northern blot results (Fig 6A), reads for AGO1-associated miR165/6 were substantially reduced in *AGO10 OE S1* as compared to wild type (Fig 6C and S9 Data). The northern blotting and sRNA-seq results demonstrated that elevated AGO10 levels allowed AGO10 to compete more effectively with AGO1 for binding to miR165/6. The only other miRNA that showed a similar effect was miR173 (Fig 6D).

We next examined whether there was any dosage effect of *AGO10* overexpression on the sequestration of miRNAs from AGO1. We conducted AGO1 IP from *AGO10 OE S2*, *M1*, *M2*, and *W1* lines and sequenced AGO1-associated small RNAs (one biological replicate; S10 Data). Indeed, there was an AGO10 dosage-dependent reduction of miR165/6 levels in AGO1 IP (Fig 6E). A similar dosage effect was found for miR173 but not for other miRNAs (Fig 6F).

### AGO10 RISCs contain higher levels of 3′ truncated miRNAs than AGO1 RISCs

**Fig 7:**
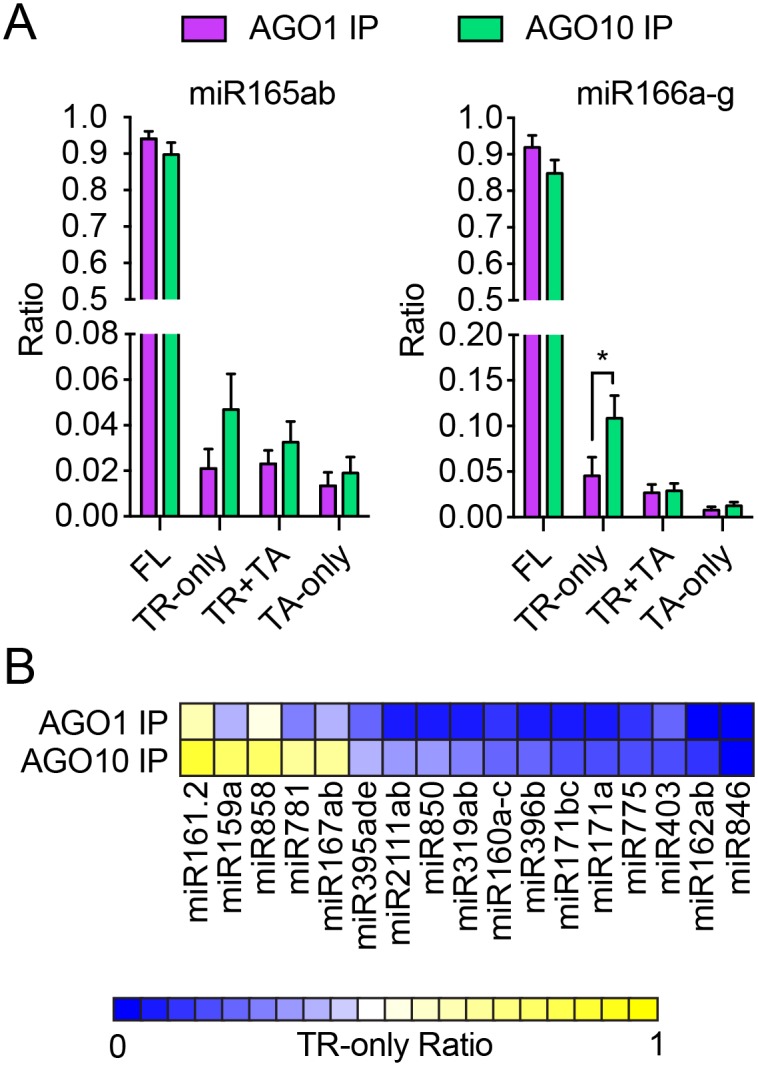
3′ truncated miRNAs in *AGO10 OE* are preferentially bound by AGO10. AGO1 and AGO10 were separately immunoprecipitated from *AGO10 OE S1*, and the associated small RNAs were subjected to high-throughput sequencing. Three biological replicates were performed, and the error bars represent standard deviations. * *p* < 0.05. (A) Proportions of various types of miR165/6 species in AGO1 IP and AGO10 IP. For miR165ab, the increase in TR-only species had a *p*-value of 0.065. (B) The TR-only ratio of various miRNAs in AGO1 IP and AGO10 IP. The TR-only ratio is the proportion of TR-only species in total species for each miRNA. Seventeen of the top 48 most abundant miRNAs are shown; they were the only ones with statistically significant differences in the TR-only ratio in AGO1 IP and AGO10 IP. All showed reduced TR-only species in AGO10 IP except for miR403. Underlying data can be found in the GEO database as series GSE58138. The raw data for panels (A and B) can be found in S1 Data file.

Mechanistically, the enhanced degradation of miR165/6 by AGO10 could happen under several scenarios. First, 3′ truncation may happen more easily when miR165/6 is bound by AGO10 than when it is bound by AGO1. Second, miR165/6 is dislodged faster from the AGO10 RISC than from the AGO1 RISC, and the degradation happens after miR165/6 is released from RISC. A third (and hybrid) model is that the initial 3′ truncation occurs on AGO10 RISC and triggers the dissociation of miR165/6 from AGO10. In the first and the third model, we would expect the AGO10 RISC to contain more 3′ truncated miR165/6 than the AGO1 RISC. Under the second scenario, AGO1 and AGO10 RISCs are not expected to differ in terms of their association with 3′ truncated species. We examined the status of 3′ tailing and 3′ truncation of miRNAs that were present in AGO1 and AGO10 IPs from *AGO10 OE S1* (sRNA-seq in three biological replicates). We found that the most abundant species of miR165/6 in both AGO1 and AGO10 RISCs were the FL miRNA (Fig 7A and S8 Data). However, the AGO10 IP showed a statistically significant increase in the TR-only miR166 species (Fig 7A). An increase in the TR-only miR165 species was also found in AGO10 IP, although the increase did not pass the *p*-value cutoff (Fig 7A). These data were consistent with the first or the hybrid model and suggested that 3′ truncation occurred, at least initially, on AGO10-associated miR165/6.

Interestingly, we observed a statistically significant increase in the levels of 3′ TR-only species in AGO10 IP for 16 out of 48 miRNAs at >1 RPM in any of the six libraries (Fig 7B and S8 Data). Only one miRNA (miR403) showed a significant reduction in 3′ TR-only species in AGO10 IP (Fig 7B). This implies that AGO10 RISCs with many different resident miRNAs are more susceptible to miRNA 3′ truncation in vivo. The lack of an effect of *AGO10* overexpression on the levels of most miRNAs is probably because these miRNAs are still mostly bound by AGO1 in *AGO10 OE*.

### AGO10-bound miR165/6 species are more susceptible to SDN1-mediated 3′ truncation in vitro

To biochemically test whether AGO10 renders miR165/6 more susceptible to 3′ truncation, we conducted SDN1 assays in vitro. An *ago10-3 His-Flag-AGO10* line [5] was used to immunoprecipitate AGO10 with anti-Flag antibodies and AGO1 with anti-AGO1 antibodies. Both IPs were successful as shown by western blotting to detect AGO1 and AGO10 as well as northern blotting to detect miR165/6 (S3A and S3C Fig). Like for AGO1 IP, SDN1 was unable to degrade miR165/6 in AGO10 IP, as shown by northern blotting to detect miR165/6 before and after incubation with SDN1 under enzyme excess conditions (S3B Fig). The lack of a large amount of AGO10 IP precluded the detection of miR165/6 3′ truncation by northern blotting (S3B Fig). We resorted to sRNA-seq to compare the degree of SDN1-mediated truncation of miR165/6 in AGO1 IP and AGO10 IP. The AGO1 IP and AGO10 IP were incubated with buffer alone (mock), SDN1, or SDN1^D283A^. After the reactions, the AGOs were precipitated, and small RNAs were isolated and subjected to high throughput sequencing (S4 Data). Two biological replicates gave reproducible results (S4 Fig). Among 13 miRNAs present at >10 RPM in AGO10 IP (in all six samples of mock, SDN1, and SDN1^D283A^), five species, including miR165/6, showed 3′ truncation by SDN1 (Fig 2B). The AGO10 IP showed more pronounced miR165/6 3′ truncation than AGO1 IP (Fig 2C and 2D), indicating that AGO10 rendered miR165/6 more susceptible to 3′ truncation by SDN1 than AGO1.

### AGO10-induced 3′ truncation of miR165/6 in vivo requires *SDN1* and *SDN2*

**Fig 8:**
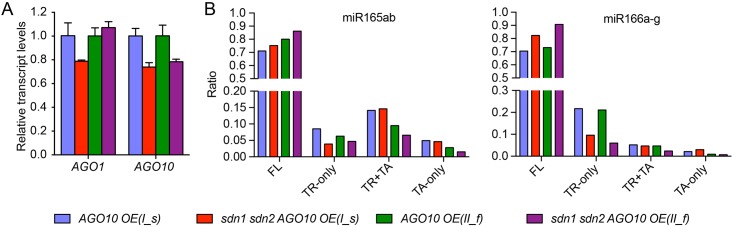
*AGO10* overexpression-induced miR165/6 3′ truncation requires *SDN1* and *SDN2*. (A) Relative transcript levels of *AGO1* and *AGO10* in Col, *AGO10 OE*, and *sdn1 sdn2 AGO10 OE* plants. *35S*::*YFP-AGO10* was introduced into wild type and *sdn1 sdn2* by transformation. Multiple T1 transgenic lines were screened for *AGO10* expression by real-time RT-PCR, and two independent pairs of lines (I and II) with similar *AGO1* and *AGO10* transcript levels were chosen for further analysis. For pair I, seedling tissues were used for the analyses (denoted by “I_s”). For pair II, inflorescence tissues were used (denoted by “II_f”). (B) The composition of the four types (FL, TR-only, TA-only, TR+TA) of reads of miR165 and miR166 in *AGO10 OE* versus *sdn1 sdn2 AGO10 OE*. Note that the proportion of 3′ TR-only reads was reduced and that of the FL reads was concordantly increased in *sdn1 sdn2* background. Although the data shown here are from one biological replicate, the two pairs of samples represented two independent experiments and showed a similar trend. Underlying data can be found in the GEO database as series GSE58138. The raw data for panels (A and B) can be found in S1 Data.

SDN1 and SDN2 mediate the 3′ truncation of some miRNAs including miR165/6 in the *hen1* background and the 3′ truncation of miR165/6 in *HEN1* backgrounds. This, together with the finding that SDN1 trimmed AGO10-bound miR165/6 in vitro, prompted us to test whether the increase in miR165/6 3′ truncation in *AGO10 OE* was mediated by SDNs. We generated a large population of primary transformants of *35S*::*YFP-AGO10* in *sdn1-1 sdn2-1* (hereafter referred to as *sdn1 sdn2*) and Col (wild type) backgrounds, identified lines that had comparable levels of *AGO10* expression in the two genotypes, (Fig 8A) and performed sRNA-seq. Sequencing small RNAs from seedlings of one pair of lines (*AGO10 OE* and *sdn1 sdn2 AGO10 OE*) or inflorescences of another independent pair showed that increased 3′ truncation of miR165/6 in *AGO10 OE* was largely suppressed by *sdn1 sdn2* (Fig 8B, S7 Fig, and S11 Data). Therefore, *AGO10* overexpression triggered SDN-dependent 3′ truncation of miR165/6. The incomplete suppression of 3′ truncation of miR165/6 by *sdn1 sdn2* was either due to the activities of other *SDN* family members or yet unknown nucleases that turnover miR165/6.

We also evaluated the effects of the *sdn* mutations on the severity of the developmental phenotypes caused by *AGO10* overexpression. The percentage of primary transformants in each of the four phenotypic categories was documented and compared between wild type and *sdn1 sdn2* (Table 1). The *sdn1 sdn2* background had higher ratios of plants with wild type—like and weak phenotypes (36.8% and 43.9%), in contrast to the low ratios in the Col background (25.7% and 11.4%; Table 1). The Col background had a higher ratio of plants with strong phenotypes as compared to that in the *sdn1 sdn2* background (48.6% versus 8.8%; Table 1). In conclusion, the degradation of miR165/6 triggered by *AGO10* overexpression and the associated developmental consequences require *SDN1* and *SDN2*.

## Discussion

### A model of miRNA degradation in vivo

Universal small RNA decay processes in plants and metazoans appear to include 3′-to-5′ truncation and 3′ uridylation. In *Arabidopsis*, the nucleotidyl transferases HESO1 and URT1 are responsible for miRNA uridylation when miRNAs lack 2′-*O*-methylation, but the enzymes responsible for miRNA 3′ truncation were unknown, and the relationship between 3′ truncation and 3′ tailing was also unknown. In this study, we provided genetic evidence documenting a role of SDN1 and SDN2 in miRNA 3′ truncation in vivo. In a *hen1* background, loss of function in *SDN1* and *SDN2* resulted in a reduction in miRNA 3′ truncation for some miRNAs. The lack of an effect on other miRNAs could be due to the presence of other *SDN* paralogs or other exonucleases. In addition, we observed that, in the *hen1 heso1* background, 3′ truncated miRNAs accumulate at much higher levels than in the *hen1* background. This supports the model (S2 Fig) that miRNA degradation is initiated by SDN-mediated 3′ truncation and followed by the uridylation of truncated species, which are further degraded by as yet unknown nucleases. Therefore, these genetic studies not only establish SDNs as one class of enzymes that causes miRNA 3′ truncation but also elucidate the relationship between miRNA 3′ truncation and 3′ tailing in miRNA turnover.

While the observations discussed above were made in the *hen1* background, genetic evidence also supports a role of SDN1 and SDN2 in miRNA 3′ truncation in the wild-type background. In the wild-type background, loss of function in *SDN1* and *SDN2* resulted in a reduction in the levels of TR-only and TR+TA miR165/6. In *AGO10 OE* plants, loss of function in *SDN1* and *SDN2* reduced the levels of 3′ truncated miR165/6 and partially rescued the developmental abnormalities. These observations were especially important, as they suggest that SDN1 and SDN2 cause the 3′ trimming of miR165/6 when it is methylated (as miRNAs are nearly completely methylated in *HEN1* backgrounds).

Furthermore, we provided biochemical evidence showing that SDN1 acts on AGO1-bound and methylated miRNAs in vitro. In fact, SDN1 had different effects on free and AGO1-bound miRNAs—it nearly completely degrades free miRNAs [30] but causes the truncation of a small and varying number of nucleotides from AGO1-bound miRNAs (this study). The 3′ truncated, AGO1-bound miRNAs caused by SDN1 in vitro mimic the 3′ truncated species in *hen1* mutants in vivo. This indicates that the 3′ trimmed miRNA species that accumulate in vivo result from the balancing act between AGO1-mediated protection and SDN1-mediated truncation.

In summary, these genetic and biochemical observations support the following model of miRNA degradation. SDNs initiate miRNA degradation in wild type by 3′ truncation of AGO1-bound and methylated miRNAs to result in AGO1-bound, 3′ truncated-and-unmethylated miRNAs, which are uridylated by HESO1 and/or URT1. The AGO1-bound, truncated-and-uridylated miRNAs are further degraded by an as yet unknown enzyme. However, we acknowledge that SDNs may not be the only exonucleases causing miRNA 3′ truncation. In addition, miRNA degradation may also entail mechanisms other than 3′ truncation and 3′ tailing.

### AGO10 destabilizes its associated miR165/6

In addition to establishing a model of miRNA degradation, the study also uncovered an unexpected function of an AGO protein in destabilizing miR165/6. In vivo, *AGO10* overexpression caused further sequestration of miR165/6 from AGO1, enhanced its 3′ truncation through SDN1/2, and reduced its accumulation. In vitro, AGO10-bound miR165/6 species were more susceptible to SDN1-mediated 3′ truncation than AGO1-bound miR165/6. The 3′ truncation of an AGO-bound miRNA should entail the displacement of the miRNA 3′ end from the binding pocket in the Piwi/Argonaute/Zwille domain [35]. Perhaps the 3′ end of miR165/6 is more accessible in an AGO10 RISC than in an AGO1 RISC. It is not known whether AGO10 confers 3′ end accessibility to its resident miRNAs in general. One observation consistent with this notion is that many miRNAs have higher levels of 3′ truncation in AGO10 IP than in AGO1 IP (Fig 7B). However, *AGO10* overexpression did not affect the abundance of most miRNAs. This is probably because most miRNAs are still bound by AGO1 despite *AGO10* overexpression (Fig 6B).

Another miRNA that is reduced in abundance by *AGO10* overexpression is miR173 (Fig 4B). *AGO10* overexpression caused a depletion of miR173 from AGO1 RISC relative to other miRNAs (Fig 6D) but not relative to AGO1 levels (Fig 6A). Thus, the reduced abundance of miR173 by *AGO10* overexpression was perhaps attributable to the lower levels of AGO1. However, increasing AGO1 levels in *AGO10 OE S1* could not restore miR173 accumulation (Fig 4C). Intriguingly, miR173 happens to be the second most preferred miRNA by AGO10 for binding in a previous study [5]. Therefore, it is likely that *AGO10* overexpression allowed AGO10 to better compete with AGO1 for binding to miR173 and lead to its degradation.

This study, together with previous studies demonstrating the importance of *AGO10*-mediated repression of miR165/6 in meristem homeostasis [1,2,5], provides an example of active miRNA degradation being employed as a mechanism to regulate stem cells in development. In developing seedlings, the spatial pattern of AGO10 protein accumulation is complementary to that of miR165/6. We propose that *AGO10* enhances the degradation of miR165/6 to help restrict this miRNA to cells not expressing *AGO10*. The clearance of miR165/6 from the SAM by *AGO10* is crucial for stem cell maintenance, as *ago10* mutants accumulate ectopic miR165/6 in the SAM and fail to maintain the stem cell population [2]. But why is such a mechanism employed to clear miR165/6 from the SAM in addition to restricting the transcription of *MIR165/6* from the stem cells? This may have to do with the potential movement of this miRNA between cells. miR165/6 probably moves across a few cell layers from its site of synthesis in the root and in leaf primordia [36–41]. The non-cell autonomy means that cell type-specific transcription alone is not sufficient to restrict the miRNA from the SAM.

## Materials and methods

### Plant materials and growth conditions

The *pnh-2* and *ago10-13* alleles [1,3] are in the Landsberg *erecta* (L*er*) background. The *hen1-8* allele and the *sdn1-1 sdn2-1* double mutant are both in the Col background and were previously described [30,42]. The *hen1-8 sdn1-1 sdn2-1* triple mutant was generated through a cross between *hen1-8* and *sdn1-1 sdn2-1*. *zll-1 ZLLp*::*YFP-ZLL* is a transgenic line in which the *YFP-ZLL* (*AGO10*) transgene driven by the *ZLL* (*AGO10*) promoter fully rescues the morphological defects of *zll-1* [11]. The *ago10-3 His-Flag-AGO10* line is in the Col background and is described [5]. Wild-type Columbia (Col) or *sdn1-1 sdn2-1* plants were transformed with the *35S*::*YFP-AGO10* plasmid via the floral dipping method [43] to obtain *AGO10 OE*. The *4mAGO1* plasmid was obtained from Dr. Herve Vaucheret (INRA, Versailles, France) and introduced into *AGO10 OE* plants via the floral dipping method.

When not specified, the plant materials used in this study were 12- to 13-d-old seedlings grown at 22°C under long day (16 hr light/ 8 hr darkness) conditions. In only one instance (mentioned in the text), inflorescences were used for small RNA sequencing in one pair of *AGO10 OE* and *sdn1 sdn2 AGO10 OE* lines.

### Plasmid construction

To generate the *AGO10* overexpression construct, FL *AGO10* coding region was amplified from cDNA using gene-specific primers containing sequences for TOPO reaction (S1 Table). The *AGO10* clone in the Gateway Entry vector was moved into pEarleyGate104 using LR reaction to produce *35S*::*YFP-AGO10*. The clone was sequenced to ensure the absence of mutations.

For the expression of recombinant SDN1 protein, the FL *SDN1* cDNA was amplified using primers SDN1 F and SDN1 R (S1 Table) and cloned into pET28-SMT3 (pSUMO). The D283A mutation was introduced into *SDN1* using the Stratagene QuikChange Site-Directed Mutagenesis Kit with a pair of primers, SDN1D283A F and SDN1D283A R (S1 Table). The pSUMO-SDN1^D283A^ clone was validated by sequencing.

### Protein expression and enzymatic assay

The pSUMO-SDN1 and pSUMO-SDN1^D283A^ plasmids were transformed into the *Escherichia coli* strain BL21 Star (DE3) for protein expression. The *E*. *coli* cells were cultured at 37°C until the OD600 reached 0.6. Isopropyl β-D-1-thiogalactopyranoside (IPTG) was added to a final concentration of 0.1 mM, and the cultures were incubated at 16°C for 16 hr. Cells were collected via centrifugation, resuspended in Lysis Buffer (20 mM Tris, pH 8.0, 500 mM NaCl, 25 mM Imidazole, pH 8.0), and sonicated on ice. The lysate was centrifuged again, and the supernatant was applied to a column containing preloaded nickel beads for purification. After two washes with Lysis Buffer, the homemade 6xHis-ULP in Dilution Buffer (20 mM Tris, pH 8.0, 50 mM NaCl, 25 mM Imidazole, pH 8.0) was loaded to the column to remove the His-SUMO tag on the recombinant proteins. The free SDN1 and SDN1^D283A^ proteins were then eluted with Elution Buffer (20 mM Tris, pH 8.0, 100 mM NaCl, 25 mM Imidazole, pH 8.0).

For SDN1 enzymatic assay, AGO1 and AGO10 complexes were immunoprecipitated from *ago10-3 His-Flag-AGO10* transgenic plants using anti-AGO1 (Agrisera) and anti-Flag (Sigma-Aldrich) antibodies, respectively. One-twelfth of the IP was used for western blotting to detect AGO1 and AGO10, another 1/12 was used for northern blotting to detect miR165/6, and the remainder was resuspended in reaction buffer (50 mM Tris, pH 8.0, 150 mM NaCl, 1mM DTT, 2.5 mM MnCl_2_, 1 mM ATP). The beads in the reaction buffer were evenly split into three parts for incubation with mock, SDN1, and SDN1^D283A^. The reactions were carried out with 2.7 μM SDN1 or SDN1^D283A^ and approximately 5.3 nM and 0.6 nM of small RNAs present in AGO1 IP and AGO10 IP, respectively (see below for the estimation of sRNA concentrations). After incubation at room temperature for 1 hr, the beads were collected again for RNA extraction, followed by small RNA library construction.

For the estimation of the amount of small RNAs in AGO1 or AGO10 IP, northern blotting was performed with the IPs and a miR165 oligonucleotide standard. The amount of miR165/6 in AGO1 and AGO10 IPs was deduced by comparing the signal intensities of miR165/6 in the IPs to those of the standard. Next, the amount of all sRNAs in the AGO1 and AGO10 IPs was estimated based on the proportions of miR165/6 reads in total small RNA reads from the AGO1 and AGO10 IPs (as determined by sRNA-seq).

### RNA extraction, real-time RT-PCR, and northern blotting

Total RNA was extracted using TRI-reagent (Molecular Research Center, Inc. TR 118). For the detection of pri- and pre-miR165/6 species together, reverse transcription was performed with random primers, and real-time PCR was then performed as described [1] with gene-specific primers located within the pre-miRNAs from each locus. Values were obtained by normalizing to *UBIQUITIN5*. Northern blotting to detect miRNAs or pre-miR166a was performed as described [44,45]. Antisense DNA oligonucleotides (S1 Table) were 5′-end labeled with γ^32^P-ATP to detect miRNAs. A DNA fragment amplified from genomic DNA using primers pre-miR166a-Nb F and pre-miR166a-Nb R (S1 Table) was randomly labeled with α^32^P-dCTP for the northern blotting to detect pre-miR166a.

### β-elimination assay

β-elimination followed by northern blotting to examine the methylation status of miR165/6 was performed as described [25].

### Immunoprecipitation and western blotting

Immunoprecipitation of AGO1 and AGO10 was performed as described [28]. In brief, 1 g of 12-day-old seedlings was ground in liquid nitrogen and dissolved in 1.5 ml IP buffer. The extract from *AGO10 OE* or *zll-1 ZLLp*::*YFP-ZLL* was incubated with anti-AGO1 antibodies (Agrisera) and anti-GFP antibodies (Clontech), respectively. Then protein-antibody complexes were captured by Dynabeads-Protein-A (Life Technologies). After washes, the beads containing AGO1 or AGO10 IPs were collected for small RNA analysis.

Western blotting to determine AGO1 protein levels was performed with anti-AGO1 antibodies, and HSC70 (Enzo Life Sciences) was used as an internal control. The His-Flag-AGO10 protein was detected using anti-AGO10 antibodies (Agrisera).

### Small RNA library construction and bioinformatics analysis

Small RNA libraries were prepared using the Illumina Tru-Seq kit [29] and the NEBNext Multiplex Small RNA Library Prep Set for Illumina kit (NEB) and sequenced with Illumina's HiSeq2000 or Illumina NextSeq500 platform at the UCR Institute for Integrative Genome Biology (IIGB) genomic core facility. Bioinformatic analyses to categorize miRNA reads into the “FL,” “TR-only,” “TA-only,” and “TR+TA” categories were performed as described [29].

### Data deposition

All large-scale sequencing datasets generated in this study and a publicly available dataset used in this study are available in the Gene Expression Omnibus (GEO) database under series GSE35479 (public dataset), GSE58138 (this study), and GSE87355 (this study).

GEO GSE35479 (public dataset)

sRNA-seq of *hen1-8* and *hen1-8 heso1-1* (two replicates each)

GEO GSE58138 (this study)

sRNA-seq of Col (wild type), *sdn1-1 sdn2-1*, *hen1-8*, *hen1-8 sdn1-1 sdn2-1* (two replicates)

sRNA-seq of AGO1 IP from Col (wild type) and *AGO10 OE* (one replicate)

sRNA-seq of AGO1 IP and AGO10 IP from Col (wild type) (three replicates)

sRNA-seq of Col (three replicates), *AGO10 OE* (three replicates), and *AGO10 OE 4mAGO1* (one replicate)

sRNA-seq of *AGO10 OE* (seedling (*I_s*)), *sdn1 sdn2 AGO10 OE* (seedling (*I_s*)), *AGO10 OE* (inflorescence (*II_f*)), and *sdn1 sdn2 AGO10 OE* (inflorescence (*II_f*)) (one replicate)

GEO GSE87355 (this study)

sRNA-seq of *AGO10 OE* lines with varying levels of *AGO10* expression (Col, *S2*, *M1*, *M2*, *W1*) (one replicate)

sRNA-seq of AGO1 IP from *AGO10 OE* lines with varying levels of *AGO10* expression (*S2*, *M1*, *M2*, *W1*) (one replicate)

sRNA-seq of AGO1 IP treated with mock, SDN1 or SDN1^D283A^ (two replicates)

sRNA-seq of AGO10 IP treated with mock, SDN1 or SDN1^D283A^ (two replicates)

## Supporting information

S1 Fig Matrices representing the compositions of reads for various miRNAs in *hen1* and *hen1 sdn1 sdn2* libraries. The X axis represents the number of nucleotides truncated from the 3′ end. The Y axis represents the number of nucleotides added to the 3′ end. The relative proportions of the species are indicated by the sizes of the circles. Two biological replicates (br1 and br2) are shown separately. Selected miRNAs with or without a reduction in 3′ truncation in *hen1 sdn1 sdn2* from Fig 1A are shown. Underlying data can be found in the GEO database as series GSE58138.
(TIF)

S2 Fig A model of miRNA degradation. SDNs initiate degradation by trimming the miRNA to result in a 3′ truncated and unmethylated miRNA, which is uridylated by HESO1 or URT1. The tailed species are further degraded by an as yet unknown enzyme. SDN1 (this study) and the nucleotidyl transferases (HESO1 and URT1) can act on AGO1-bound miRNAs as well as free miRNAs.
(TIF)

S3 Fig SDN1 enzymatic assays with AGO1 and AGO10 immunoprecipitates as substrates. Immunoprecipitation (IP) was performed with wild type (Col) or a line with the *His-Flag-AGO10* transgene in an *ago10* mutant background [5]. AGO1 and AGO10 IP was performed with anti-AGO1 and anti-Flag antibodies, respectively. (A) The AGO1 IP was subjected to western blotting to detect AGO1 and northern blotting to detect miR165/6. (B) SDN1 enzymatic assays with AGO1 IP, AGO10 IP, and an RNA oligonucleotide as substrates under enzyme excess conditions. Northern blotting was performed to detect miR165/6 in the reactions with AGO1 and AGO10 IPs as substrates. The RNA oligonucleotide was 5′ labeled with ^32^P to aid detection. The bands below the full-length form were shorter species present in the RNA oligonucleotide preparation. These shorter versions as well as the full-length form were degraded by SDN1. (C) AGO10 IP was subjected to western blotting with anti-AGO10 antibodies to detect AGO10 and northern blotting to detect miR165/6. The band present in the transgenic line but not in Col is His-Flag-AGO10. The other bands in the input samples are likely non-specific signals.
(TIF)

S4 Fig Hierarchical clustering analysis showing the degree of similarity among the sRNA-seq libraries. Sample-to-sample distances were calculated based on log-transformed normalized read counts. The biological replicates of each sample type were highly reproducible. The raw data can be found in S1 Data file.
(TIF)

S5 Fig Characterization of *AGO10* over expression lines. (A) Real-time RT-PCR to quantify transcript levels of *AGO1* and *AGO10* in wild type (Col) and *AGO10 OE S1*. ** p-value < 0.01, *** p-value < 0.001. (B) Real-time RT-PCR to detect pri/pre-miR165/6 from eight *MIR165*/*6* genes that were expressed in seedlings. (C) Northern blotting to detect pre-miR166a in the indicated genotypes. *pnh-2* is an *ago10* mutant in the L*er* background. *AGO10 OE S1* and *pnh-2 AGO10 OE* are two independent *AGO10 OE* lines in Col and Ler accessions, respectively. The stained gel is shown on the left. U6 was an internal control. (D) Western blotting to determine AGO1 levels in wild type (Col) and *AGO10 OE S1*. Three biological replicates were performed. The numbers represent AGO1 levels relative to wild type in each replicate. HSC70 was the loading control. (E) Real-time RT-PCR to determine *AGO1* and *AGO10* transcript levels in *AGO10 OE S1* and *AGO10 OE 4mAGO1*. (F) Western blotting to detect AGO1 in the indicated genotypes. AGO1 protein levels were increased in *AGO10 OE 4mAGO1*. HSC70 was the loading control. Underlying data can be found in the GEO database as series GSE58138. The raw data for panels (A, B and E) can be found in S1 Data file.
(TIF)

S6 Fig Matrices representing the compositions of miR165/6 reads in Col, *AGO10 OE S1* and *AGO10 OE 4mAGO1* libraries. miR165/6 3′ truncation was increased in *AGO10 OE S1* relative to wild type. *4mAGO1* failed to rescue this increase in miR165/6 3′ truncation. The data were based on one biological replicate. Underlying data can be found in the GEO database as series GSE58138.
(TIF)

S7 Fig Matrices representing the compositions of miR165/6 reads in *AGO10 OE* and *sdn1 sdn2 AGO10 OE* libraries. Total small RNAs were sequenced from seedling tissues (“s”) of one pair (I) of transgenic lines (*AGO10 OE* and *sdn1 sdn2 AGO10 OE*) and inflorescence tissues (“f”) of another independent pair (II) of transgenic lines. *AGO10* over expression in the *sdn1 sdn2* double mutant caused lower levels of miR165/6 3′ truncation. Although the data were based on a single biological replicate, the two independent pairs served as experimental repeats and gave similar trends. Underlying data can be found in the GEO database as series GSE58138.
(TIF)

S1 Table Oligonucleotides used in this study. (DOCX)

S1 Data Raw data for all figures and supplemental figures. (XLSX)

S2 Data Composition of miRNAs in Col, *sdn1 sdn2*, *hen1*, and *hen1 sdn1 sdn2*. (XLSX)

S3 Data Composition of miRNAs in *hen1* and *hen1 heso1*. (XLSX)

S4 Data Composition of miRNA forms in AGO1 IP and AGO10 IP treated with mock (no enzyme), SDN1, and SDN1 catalytic mutant (SDN1^D283A^). (XLSX)

S5 Data Composition of miRNA forms in Col and various *AGO10 OE* lines (*S2*, *M1*, *M2*, and *W1*). (XLSX)

S6 Data Composition of miRNA forms in Col and *AGO10 OE S1*. (XLSX)

S7 Data Composition of miRNA forms in Col, *AGO10 OE S1*, and *AGO10 OE 4mAGO1*. (XLSX)

S8 Data Profile of AGO1- and AGO10-bound miRNAs in *AGO10 OE S1*. (XLSX)

S9 Data Profile of AGO1-bound miRNAs in Col and *AGO10 OE S1*. (XLSX)

S10 Data Composition of miRNA forms in AGO1 IP from various *AGO10 OE* lines (*S2*, *M1*, *M2*, and *W1*). (XLSX)

S11 Data Composition of miRNA forms in *AGO10 OE* and *sdn1 sdn2 AGO10 OE*. (XLSX)

## Abbreviations

AGO: ARGONAUTE
*AGO10*: *ARGONAUTE10*
DCL1: DICER-LIKE1
FL: full-length
GEO: Gene Expression Omnibus
IP: immunoprecipate
miRNA: microRNA
piRNAs: Piwi-interacting RNAs
*PNH*: *PINHEAD*
RISC: RNA-induced silencing complex
RPM: reads per million
RT-PCR: reverse transcription PCR
SAM: shoot apical meristem
SDN: SMALL RNA DEGRADING NUCLEASE
siRNAs: small interfering RNAs
sRNA-seq: small RNA high throughput sequencing
TA-only: 3′ tailed-only
TR+TA: 3′ truncated-and-tailed
TR-only: 3′ truncated-only
*ZLL*: *ZWILLE*
