# Correction: ARGONAUTE10 promotes the degradation of miR165/6 through the SDN1 and SDN2 exonucleases in *Arabidopsis*

**DOI:** 10.1371/journal.pbio.3001120

**Published:** 2021-02-11

**Authors:** Yu Yu, Lijuan Ji, Brandon H. Le, Jixian Zhai, Jiayi Chen, Elizabeth Luscher, Lei Gao, Chunyan Liu, Xiaofeng Cao, Beixin Mo, Jinbiao Ma, Blake C. Meyers, Xuemei Chen

In the Data Deposition section of the Material and Methods, there is an error in the seventh sentence. The sentence should read: sRNA-seq of AGO1 IP and AGO10 IP from AGO10 OE (three replicates).
