# Supplementary figures and images for: ARGONAUTE10 promotes the degradation of miR165/6 through the SDN1 and SDN2 exonucleases in Arabidopsis
Source: PLoS Biol. 2017 Feb 23;15(2):e2001272. doi: 10.1371/journal.pbio.2001272 (PMC5322904; doi:10.1371/journal.pbio.2001272)

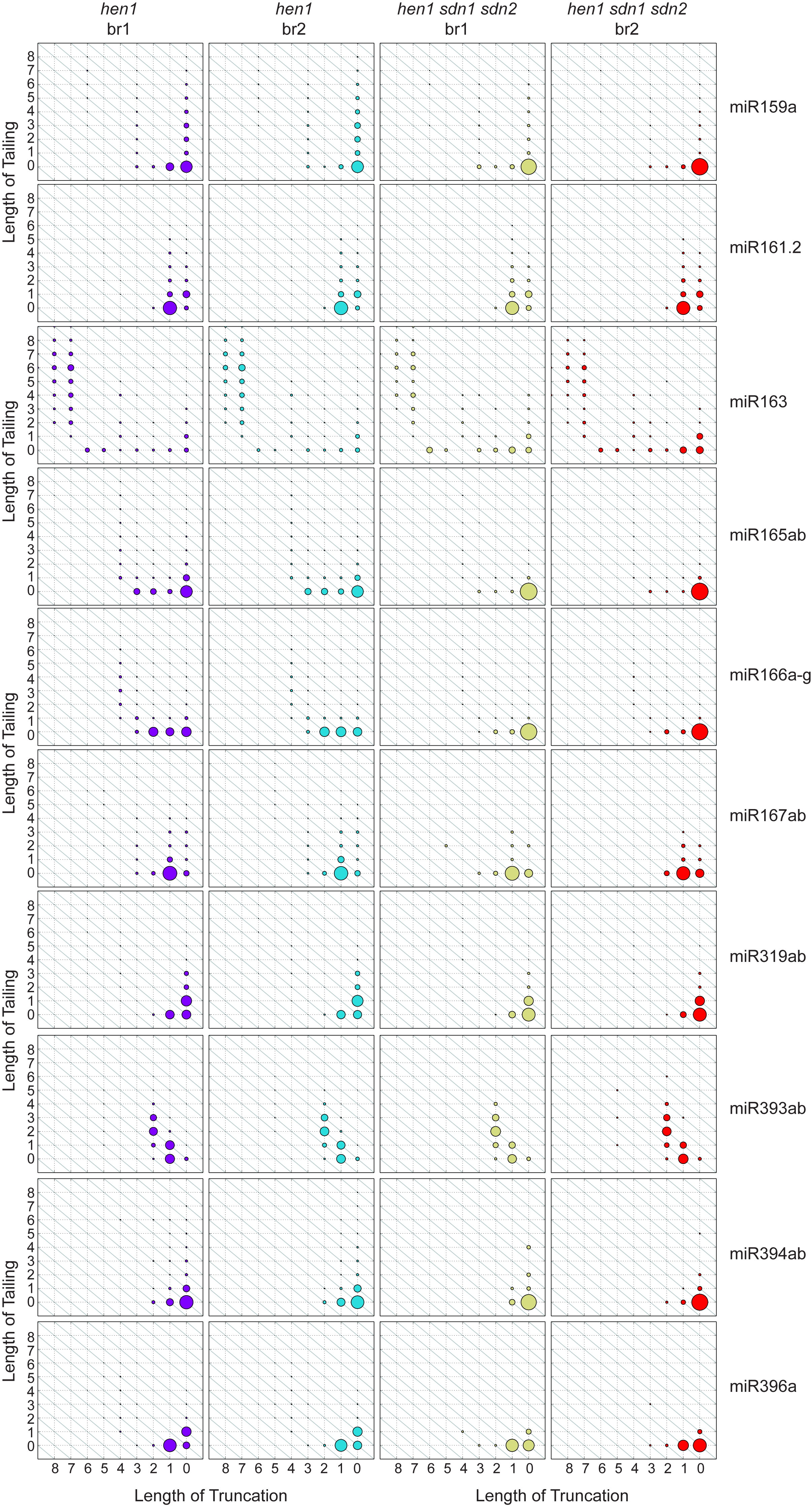

Supplement: S1 Fig — The X axis represents the number of nucleotides truncated from the 3′ end. The Y axis represents the number of nucleotides added to the 3′ end. The relative proportions of the species are indicated by the sizes of the circles. Two biological replicates (br1 and br2) are shown separately. Selected miRNAs with or without a reduction in 3′ truncation in hen1 sdn1 sdn2 from Fig 1A are shown. Underlying data can be found in the GEO database as series GSE58138. (TIF) [file pbio.2001272.s001.tif]

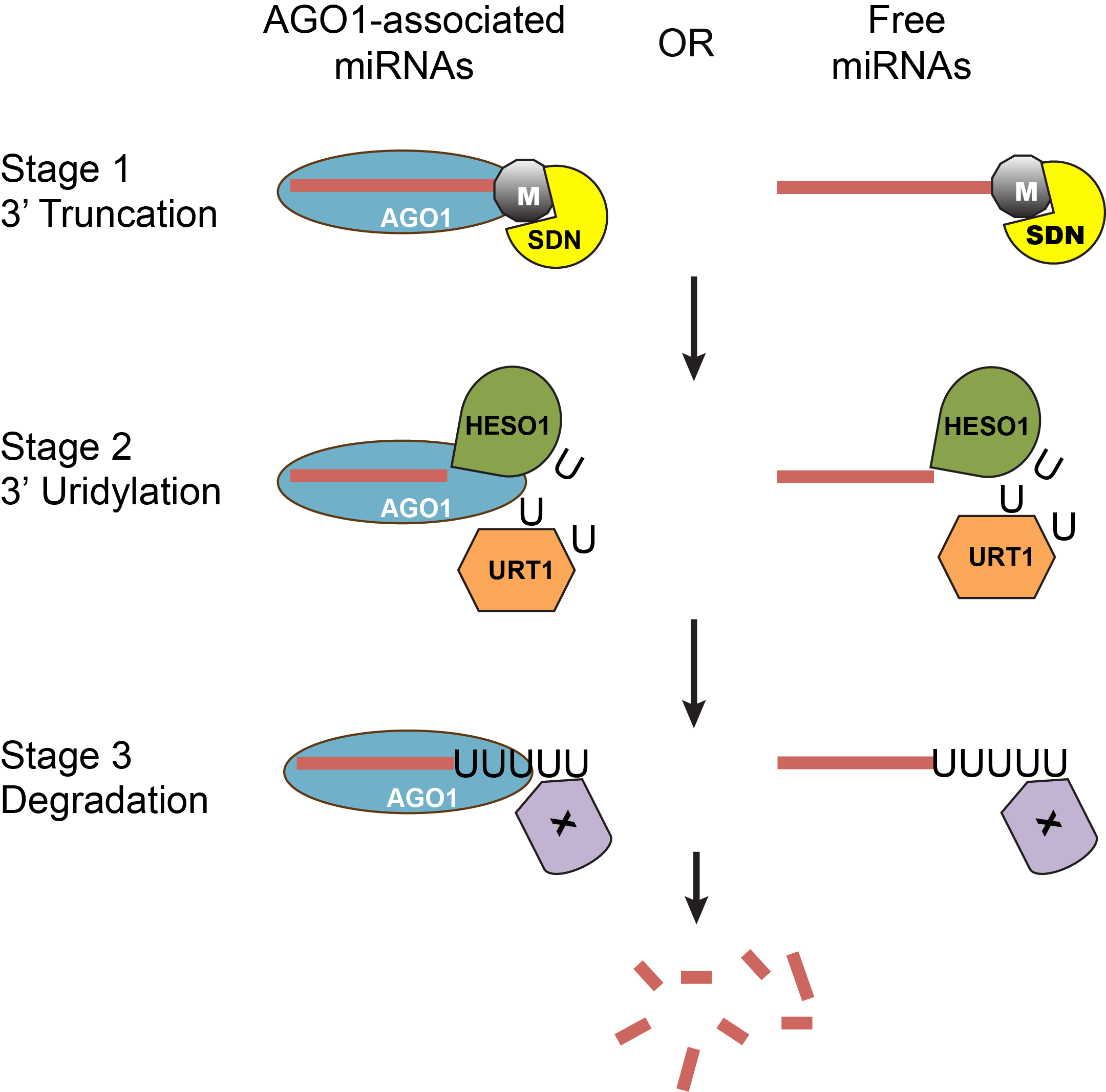

Supplement: S2 Fig — SDNs initiate degradation by trimming the miRNA to result in a 3′ truncated and unmethylated miRNA, which is uridylated by HESO1 or URT1. The tailed species are further degraded by an as yet unknown enzyme. SDN1 (this study) and the nucleotidyl transferases (HESO1 and URT1) can act on AGO1-bound miRNAs as well as free miRNAs. (TIF) [file pbio.2001272.s002.tif]

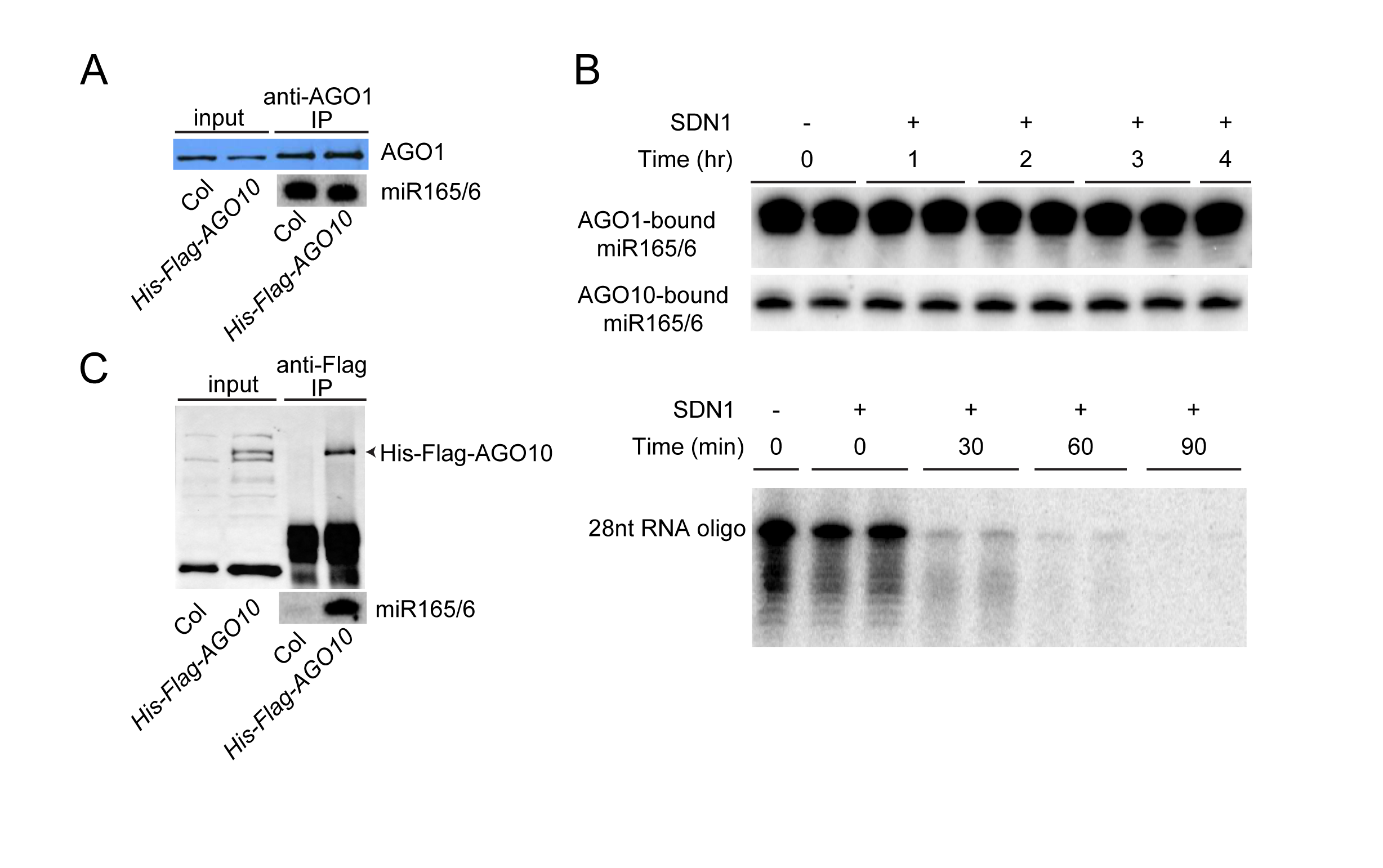

Supplement: S3 Fig — Immunoprecipitation (IP) was performed with wild type (Col) or a line with the His-Flag-AGO10 transgene in an ago10 mutant background [5]. AGO1 and AGO10 IP was performed with anti-AGO1 and anti-Flag antibodies, respectively. (A) The AGO1 IP was subjected to western blotting to detect AGO1 and northern blotting to detect miR165/6. (B) SDN1 enzymatic assays with AGO1 IP, AGO10 IP, and an RNA oligonucleotide as substrates under enzyme excess conditions. Northern blotting was performed to detect miR165/6 in the reactions with AGO1 and AGO10 IPs as substrates. The RNA oligonucleotide was 5′ labeled with 32P to aid detection. The bands below the full-length form were shorter species present in the RNA oligonucleotide preparation. These shorter versions as well as the full-length form were degraded by SDN1. (C) AGO10 IP was subjected to western blotting with anti-AGO10 antibodies to detect AGO10 and northern blotting to detect miR165/6. The band present in the transgenic line but not in Col is His-Flag-AGO10. The other bands in the input samples are likely non-specific signals. (TIF) [file pbio.2001272.s003.tif]

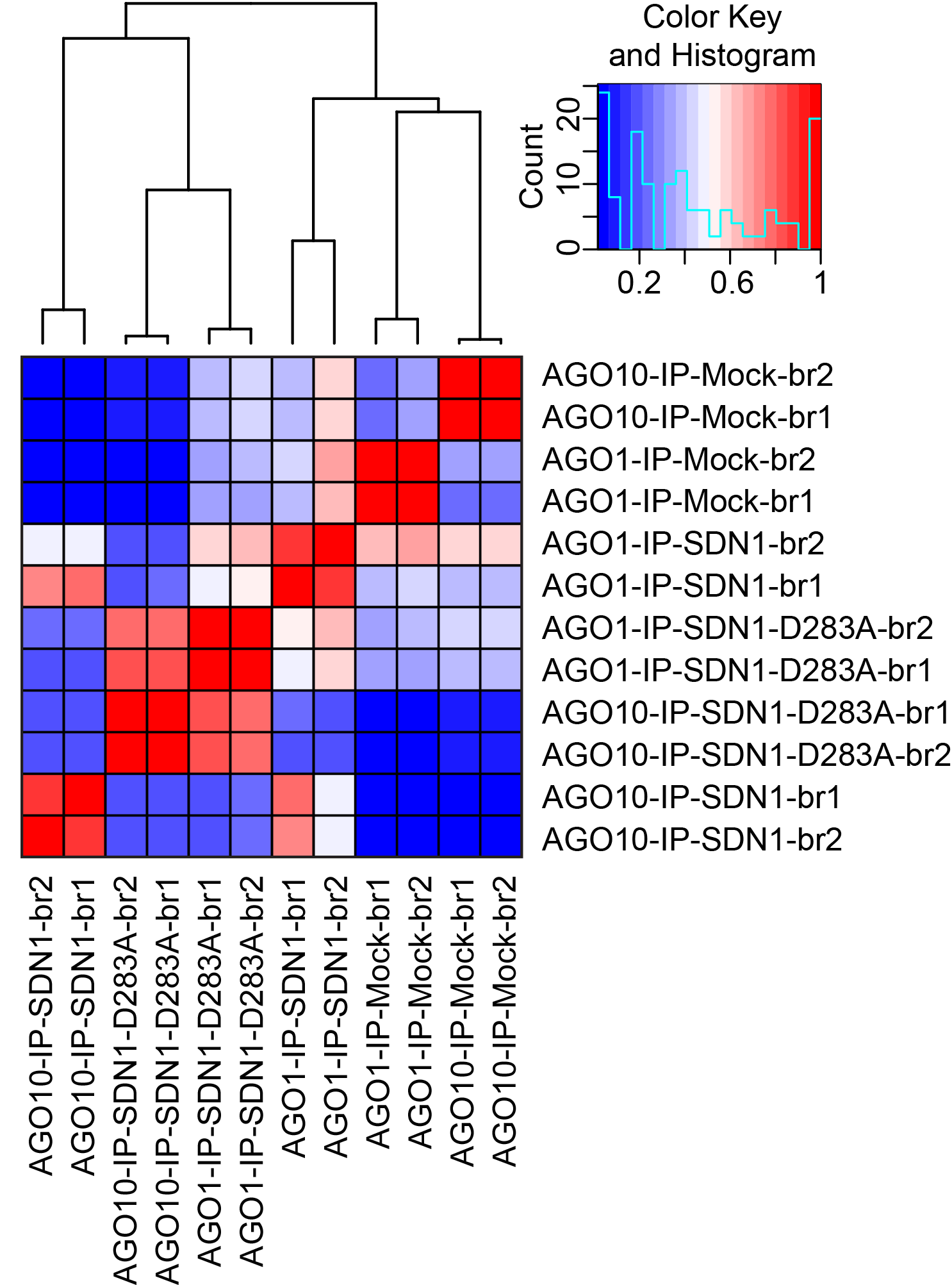

Supplement: S4 Fig — Sample-to-sample distances were calculated based on log-transformed normalized read counts. The biological replicates of each sample type were highly reproducible. The raw data can be found in S1 Data file. (TIF) [file pbio.2001272.s004.tif]

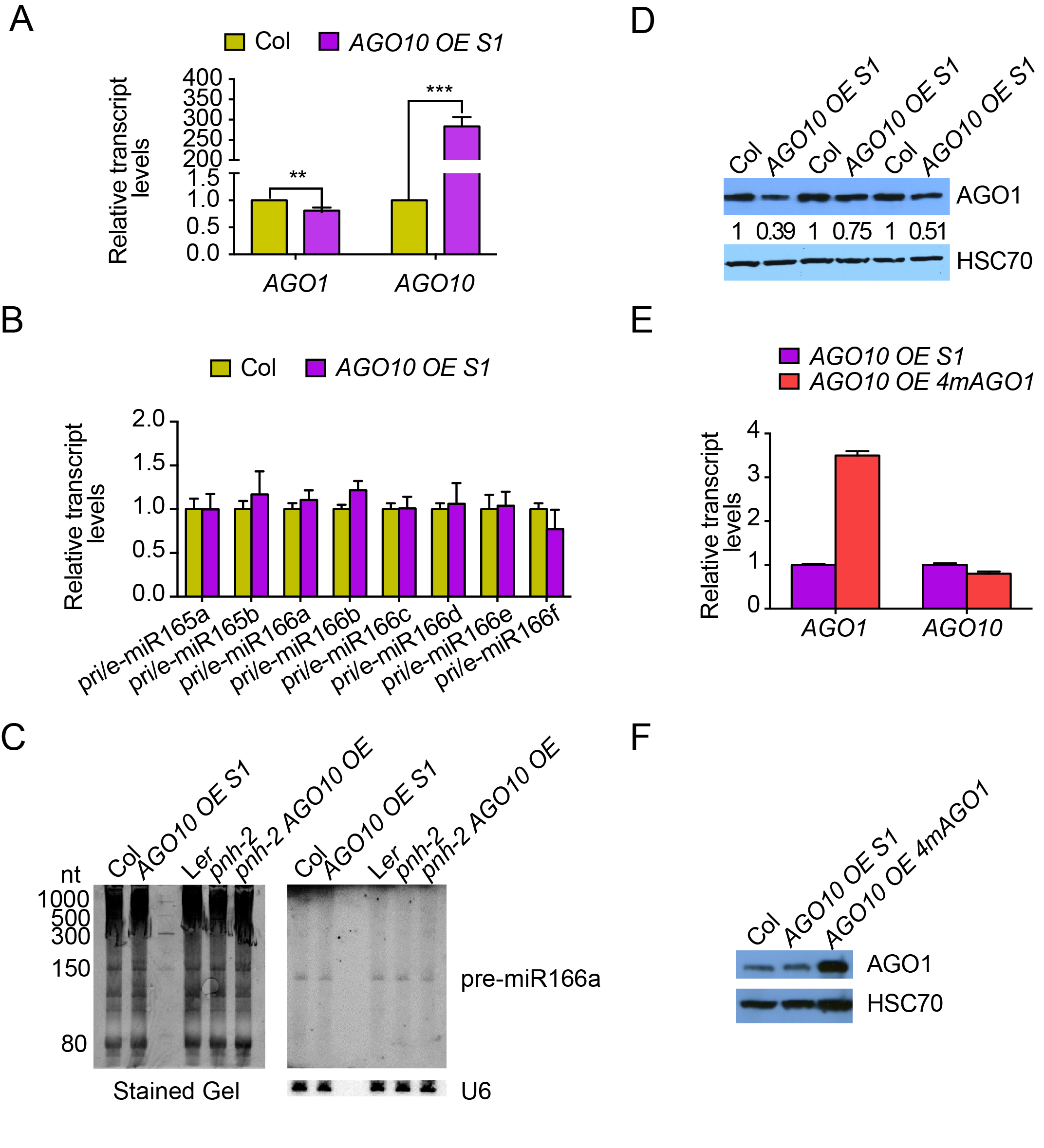

Supplement: S5 Fig — (A) Real-time RT-PCR to quantify transcript levels of AGO1 and AGO10 in wild type (Col) and AGO10 OE S1. ** p-value < 0.01, *** p-value < 0.001. (B) Real-time RT-PCR to detect pri/pre-miR165/6 from eight MIR165/6 genes that were expressed in seedlings. (C) Northern blotting to detect pre-miR166a in the indicated genotypes. pnh-2 is an ago10 mutant in the Ler background. AGO10 OE S1 and pnh-2 AGO10 OE are two independent AGO10 OE lines in Col and Ler accessions, respectively. The stained gel is shown on the left. U6 was an internal control. (D) Western blotting to determine AGO1 levels in wild type (Col) and AGO10 OE S1. Three biological replicates were performed. The numbers represent AGO1 levels relative to wild type in each replicate. HSC70 was the loading control. (E) Real-time RT-PCR to determine AGO1 and AGO10 transcript levels in AGO10 OE S1 and AGO10 OE 4mAGO1. (F) Western blotting to detect AGO1 in the indicated genotypes. AGO1 protein levels were increased in AGO10 OE 4mAGO1. HSC70 was the loading control. Underlying data can be found in the GEO database as series GSE58138. The raw data for panels (A, B and E) can be found in S1 Data file. (TIF) [file pbio.2001272.s005.tif]

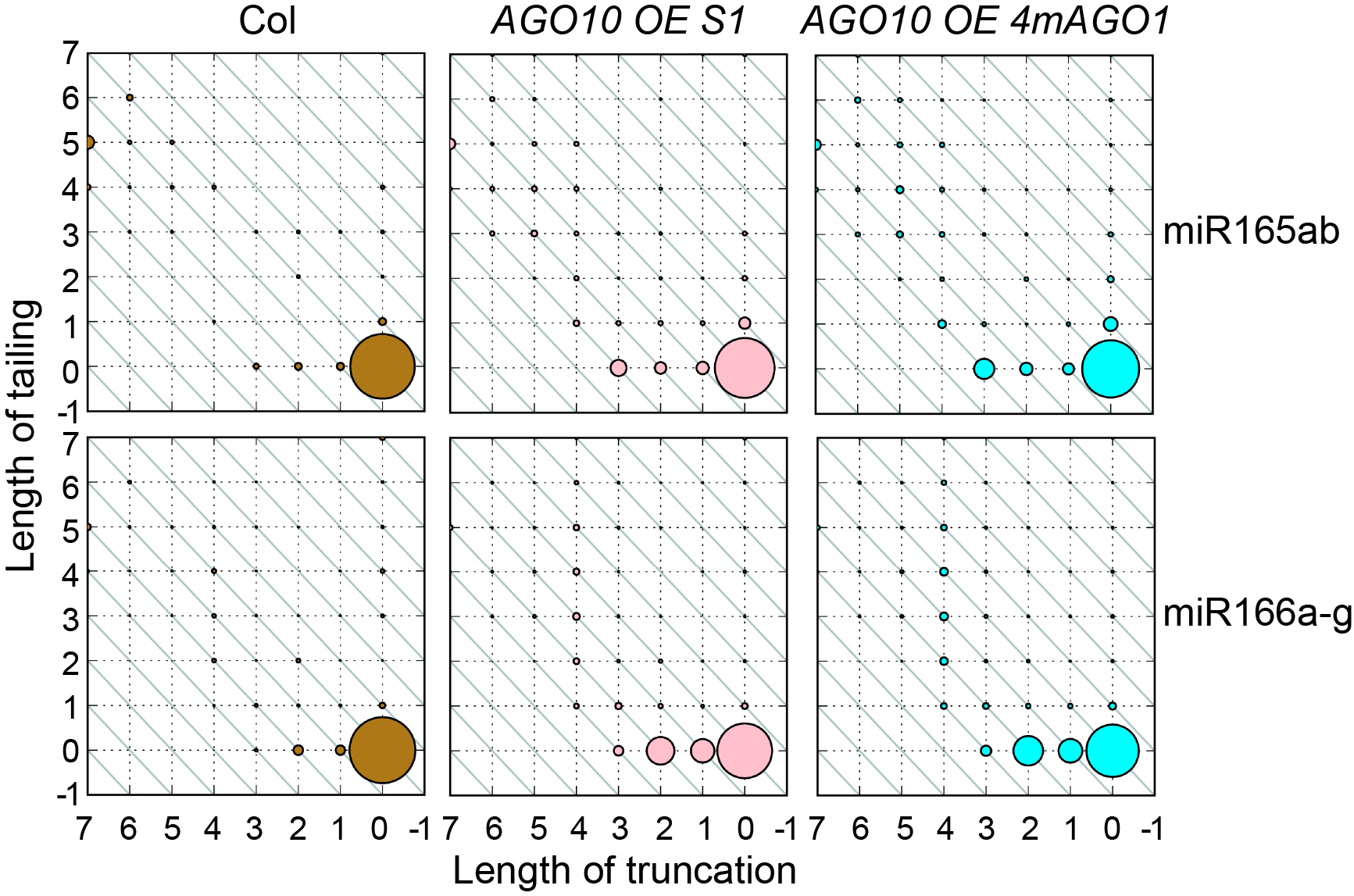

Supplement: S6 Fig — miR165/6 3′ truncation was increased in AGO10 OE S1 relative to wild type. 4mAGO1 failed to rescue this increase in miR165/6 3′ truncation. The data were based on one biological replicate. Underlying data can be found in the GEO database as series GSE58138. (TIF) [file pbio.2001272.s006.tif]

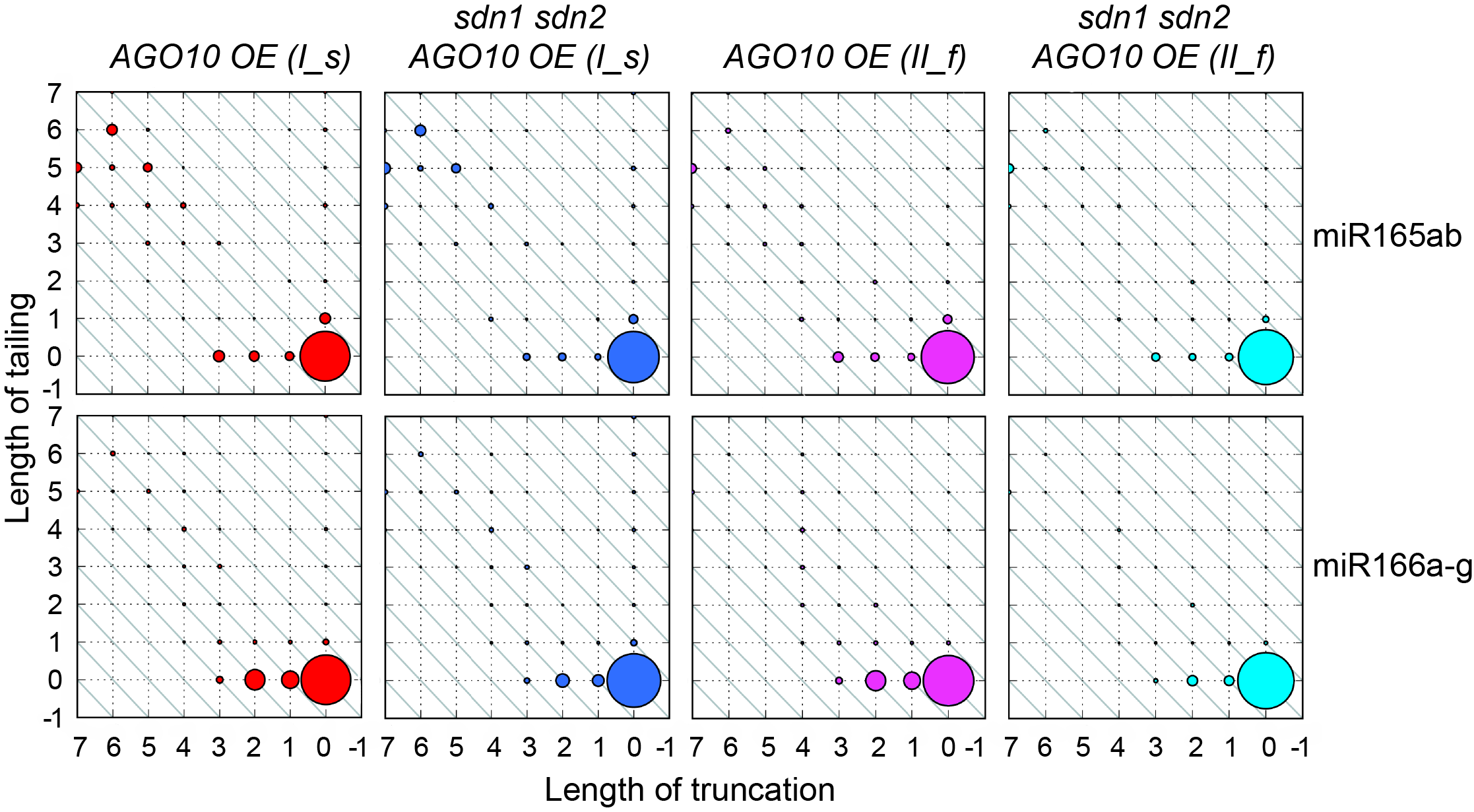

Supplement: S7 Fig — Total small RNAs were sequenced from seedling tissues (“s”) of one pair (I) of transgenic lines (AGO10 OE and sdn1 sdn2 AGO10 OE) and inflorescence tissues (“f”) of another independent pair (II) of transgenic lines. AGO10 over expression in the sdn1 sdn2 double mutant caused lower levels of miR165/6 3′ truncation. Although the data were based on a single biological replicate, the two independent pairs served as experimental repeats and gave similar trends. Underlying data can be found in the GEO database as series GSE58138. (TIF) [file pbio.2001272.s007.tif]
