# Supplementary material for: ARGONAUTE10 promotes the degradation of miR165/6 through the SDN1 and SDN2 exonucleases in Arabidopsis
Source: PLoS Biol. 2017 Feb 23;15(2):e2001272. doi: 10.1371/journal.pbio.2001272 (PMC5322904; doi:10.1371/journal.pbio.2001272)
Supplement: S1 Table — (DOCX) [file pbio.2001272.s008.docx]

S1 Table. Oligonucleotides used in this study.

| Oligo Name | Sequence |
| --- | --- |
| caccAGO10 F | caccATGCCGATTAGGCAAATGAAAGA |
| AGO10 R | TTAGCAGTAGAACATTACTCTCT |
| SDN1 F | ACGGATCCGAGCTTAAACTAGCCACCGCCGA |
| SDN1 R | ACGCGTCGACTCATTCATCTTGAACCATTTTACGGACA |
| SDN1D283A F | CATGATTGTGTACATGCCGCATCAGCTGCGATGAAACTTG |
| SDN1D283A R | CAAGTTTCATCGCAGCTGATGCGGCATGTACACAATCATG |
| UBQ5 F | GGTGCTAAGAAGAGGAAGAA |
| UBQ5 R | CTCCTTCTTTCTGGTAAACGT |
| pre-miR165a F | TTGTCTGGATCGAGGATATTATAGATAT |
| pre-miR165a R | TCCGAGGATACTCTCTATGATCACT |
| pre-miR165b F | TGTTGTTTGGATCGAGGATATCA |
| pre-miR165b R | TACCATGTGGCATGTATGTATATATATGTA |
| pre-miR166a F | TTCATGTTGGATCTCTTTCGATCT |
| pre-miR166a R | CGCTAAAACCCTAATCAAATCTG |
| pre-miR166b F | TGGCTCGAGGACTCTTATTCTAA |
| pre-miR166b R | AAACCCTAATCAATCATCAGATCTG |
| pre-miR166c F | TTGTCTGGCTCGAGGTCATG |
| pre-miR166c R | GGGTTTTCTTAATTTGTTCTTCCAAA |
| pre-miR166d F | TTGTCTGGCTCGAGGTCATGA |
| pre-miR166d R | TGGTCCGAGAATCATTTAGGG |
| pre-miR166e F | GCACGAGGCCCTTAACTTAGATC |
| pre-miR166e R | TCCGACGTCATTAACCGTAAAA |
| pre-miR166f F | GAATGATGCCTGGCTCGAGAC |
| pre-miR166f R | GGAATGAAGCCTGGTCCGACA |
| pre-miR166g F | CGAGGTCATGGAGAGTAATTCG |
| pre-miR166g R | AGCCTGGTCCGAGAATCATTT |
| AGO1-RT F | TGGACCACCGCAGAGACAAT |
| AGO1-RT R | CATCATACGCTGGAAGACGAC |
| AGO10-RT F | GGGTATTCAGGGAACAAGCA |
| AGO10-RT R | TAGAAACGTGCTCGAAATGCT |
| miR159-AS probe | TAGAGCTCCCTTCAATCCAAA |
| miR166-AS probe | GGGGaaTGaaGCCTGGTCCGa |
| miR168-AS probe | TTCCCGACCTGCACCAAGCGA |
| miR173-AS probe | GTGATTTCTCTCTGTAAGCGA |
| miR393-AS probe | GATCAATGCGATCCCTTTGGA |
| miR395-AS probe | GAGTTCCCCCAAACACTTCAG |
| U6 probe | AGGGGCCATgctaatcttctc |
| pre-miR166a-Nb F | AGATATATATTCAGAAACCCTAG |
| pre-miR166a-Nb R | GGTTCATTCACTGGATCTGAAAC |
